# Estimating flow division in aortic branches of diseased aorta: a method for boundary condition specification in CFD analysis

**DOI:** 10.3389/fbioe.2025.1640687

**Published:** 2025-11-13

**Authors:** Mengqiang Hu, Ming Yang, Zhihao Ding, Shu Chen, Xiaoyu Qi, Chuanzhi Zhu, Yining Zhang, Chao Yang, Yuanming Luo

**Affiliations:** 1 State Key Laboratory of Transvascular Implantation Devices, Hangzhou, China; 2 Department of Technology, Boea Wisdom (Hangzhou) Network Technology Co., Ltd., Hangzhou, China; 3 Department of Radiology, Union Hospital, Tongji Medical College, Huazhong University of Science and Technology, Wuhan, China; 4 Hubei Province Key Laboratory of Molecular Imaging, Wuhan, China; 5 Department of Cardiovascular Surgery, Union Hospital, Tongji Medical College, Huazhong University of Science and Technology, Wuhan, China; 6 Department of Vascular Surgery, Union Hospital Tongji Medical College, Huazhong University of Science and Technology, Wuhan, China; 7 Department of Mechanical Engineering, The University of Iowa, Iowa, IA, United States

**Keywords:** hemodynamics, windkessel model, aortic diseases, CFD, boudary condition

## Abstract

Hemodynamic predictions using computational fluid dynamics (CFD) simulations can provide valuable guidance assessing aortic disease risks. However, their reliability is hindered by the lack of patient-specific boundary conditions, particularly measured flow rates. This study addresses this knowledge gap by introducing a method for estimating flow division in aortic branches. The geometry of the lesional aorta was first repaired to obtain a near-healthy reference geometry. An iterative CFD simulation was then employed to estimate the flow division in the branches of the diseased aorta. Specifically, empirical boundary conditions from healthy individuals were used to predict the outlet pressures of reference geometry, which were subsequently converted into resistance models. These resistance models were then assigned to the outlets of the diseased aorta to predict the inlet pressure. The discrepancy between the predicted and target inlet pressures was iteratively minimized by adjusting the inlet pressure of the reference model until convergence was achieved. The final flow division in the branches of the diseased aorta was then obtained. The performance of the proposed method was investigated in three patients with aortic dissection or aneurysm. The proposed method predicted lower flow rates in branches with severe stenosis, which was more consistent with physiological expectations. Furthermore, the predicted blood pressure differed significantly from that obtained using the traditional method and was closer to the target values. The proposed method provides a practical solution for specifying boundary conditions in hemodynamic studies when clinically measured flow rates are unavailable.

## 1 Introduction

Cardiovascular diseases pose a significant threat to human health (Mensah et al., 2019). Among these, the aortic-related diseases, such as aortic aneurysm and aortic dissection (Sterpetti et al., 2024), usually causes a high mortality rate since the aorta serves as a crucial bridge supplying blood from the heart to the rest of the body. Hemodynamic investigation based on CFD can provide more integrated guidance for disease management in patients with aortic diseases (Tse et al., 2011; Bianchi et al., 2017; Hu et al., 2025).

Accurate hemodynamic predictions using CFD depend on patient-specific aortic geometry and boundary conditions (Taylor and Figueroa, 2009; Youssefi et al., 2018; Armour et al., 2022). Advances in medical imaging technology have enabled precise acquisition of patient-specific aortic geometry. Even minor branches of the aorta have been successfully reconstructed in hemodynamic studies (Stokes et al., 2023b). As for patient-specific boundary conditions, advanced technologies like 4D Flow MRI can capture multi-dimensional blood velocity (Azarine et al., 2019). However, their application is typically limited to large academic centers rather than routine clinical use (Menon et al., 2024). Idealized or simplified boundary conditions, such as constant pressure, empirical pulsatile pressure, empirical flowrate, Murray-law, and 0D lumped parameter model for outflow strategy, have been widely used (Dillon-Murphy et al., 2016; Zambrano et al., 2016; Peng et al., 2019; Zhang et al., 2023). The Windkessel model, a type of lumped parameter model, is particularly popular because it can not only achieve specific downstream flow division, but also reproduce the patient’s blood pressure at the ascending aorta inlet (Stergiopulos et al., 1999b).

The Windkessel model analogized the target vascular system to an electric circuit. For aortic hemodynamics investigations, downstream arteries, arterioles, and capillaries are typically simplified into a lumped parameter model. This model is then coupled with the 3D aortic geometry to conduct multi-scale simulations. In the electric circuit analogy, commonly used components include resistors to model blood’s viscous resistance, capacitors to model vessel wall compliance, and inductors to model blood inertia. The commonly used three-element Windkessel model comprises a characteristic impedance *R*_c_ for resistance in large arteries, a peripheral impedance *R*_p_ for resistance in distal vessels, and a capacitance *C* in parallel with *R*_p_ for the total compliance of the arterial tree (Westerhof et al., 1971).

To reproduce the ascending aorta inlet pressure and downstream branch flow division, parameters of the three-element Windkessel model should theoretically be estimated using patient-specific data. Tuning strategies for these parameters can be divided into manual “trial and error” tunning (Stergiopulos et al., 1999a; Chung, 2005; Les et al., 2010) and automatic iterative optimization tunning (Toorop et al., 1987; Spilker and Taylor, 2010; Ismail et al., 2013; Alimohammadi et al., 2014; Pant et al., 2014; Xiao et al., 2014; Bonfanti et al., 2019; Arthurs et al., 2020; Li and Mao, 2023). In manual methods, resistance is estimated based on the mean blood pressure and flow rate, while capacitance is determined using methods such as the pulse pressure method (Stergiopulos et al., 1999a), the decay time method (Laskey et al., 1990), or the area method (Liu et al., 1986). In iterative optimization methods, the objective functions are typically formulated to minimize the difference between the calculated and target inlet pressure and/or downstream flow rate. Li and Mao (Li and Mao, 2023) proposed a fast approach to obtain the parameters using a pattern search algorithm and an only-once steady-state CFD simulation; notably, the flow resistance of the 3D aortic geometry was considered in the optimization process.

The flow division in downstream branches is an essential input in the tuning process of the parameters. Unfortunately, patient-specific flow rates are usually unavailable in clinic settings. As a result, empirical flow division is widely used. Tricarico et al. (2023) proposed a method to approximate the parameters of the three-element Windkessel model. They first calculated population-averaged, artery-specific normalized parameters according to the patient-specific flow rate and pressure waveforms measured in a patient cohort. These parameters were then integrated with brachial pressure values and mean flow rates, estimated from arterial diameter, to determine the resistance and capacitance for new patients. However, representative population-averaged data relies heavily on a large patient cohort and estimating mean flow rates based on arterial diameter encounters challenges related to accuracy and variability, especially in patients with vessel lesions. In Alimohammadi et al.’s study (Alimohammadi et al., 2014), an initial steady-state CFD simulation with zero-pressure outlets was conducted to determine a flow division, which was then used to estimate the parameters of the Windkessel model using a data assimilation technique. However, using a zero-pressure outlet for predictions deviates significantly from physiological reality and results in a flow division that differs from actual values (Chnafa et al., 2018).

Aortic diseases often involve abnormal dilation or stenosis of vessels, altering downstream flow division and potentially causing severe complications such as visceral or lower limb malperfusion. For hemodynamic investigations, using empirical flow division from healthy subjects will undoubtedly yield inaccurate results. Bonfanti et al. (2019) conducted multi-scale simulations for hemodynamic analysis of three complex type-B aortic dissection (TBAD) cases, one of which involved left renal malperfusion, as evidenced by contrast-enhanced CT scans. They wisely reduced blood flow rate to the left kidney by 29% in the empirical flow division to align with the patient’s condition. However, obtaining quantitative evidence of malperfusion in other branch arteries remains challenging due to the lack of reference for contrast.

Therefore, developing a method to estimate the flow division in branches of the diseased aorta is crucial. In this study, we propose an approach to achieve this goal and facilitate the parameter estimation process of the Windkessel model. First, the geometry of the diseased aorta was repaired based on established principles to obtain a near-healthy reference geometry. Then, an iterative CFD-based framework was designed to estimate the flow division by performing simulations on both the reference and lesional geometries. Specifically, empirical boundary conditions from healthy individuals were applied to the reference geometry to conduct CFD simulations to predict the outlet pressures. These outlet pressures were subsequently converted into resistance models and incorporated into the lesional model, where additional CFD simulations were conducted to predict the inlet pressure. The discrepancy between the predicted and target inlet pressures of the diseased aorta was iteratively minimized by adjusting the inlet pressure of the reference model until convergence was achieved. At convergence, the physiological state of the lesional model was considered to best approximate the patient’s actual condition, and the resulting flow division was deemed physiologically relevant. The performance of the proposed method was evaluated through its application to three patients with aortic dissection or aneurysm.

## 2 Materials and methods

### 2.1 Patient information

Three patients (56–68–69-year-old, 1 female and 2 males) with aortic diseases from the Union Hospital, Tongji Medical College, Huazhong University of Science and Technology were included in this study. In addition to abdominal aneurysm or dissection, two patients (Patients 1 and 2) exhibited arterial stenosis at the origins of certain visceral arteries due to atherosclerosis or calcification. The third patient (Patient 3) presented with a focal narrowing of the descending aorta caused by aortic dissection. Patient details are summarized in Table 1, and more detailed anatomical measurements are available in the Supplementary Material.

**TABLE 1:** Patient information.

Item	Patient 1	Patient 2	Patient 3
Gender	Male	Male	Female
Age (year)	68	69	56
Disease characteristics	• Type: TBAD • Number of tears: 2 • PET area: ∼40.7 mm^2^ • TL volume: 125.5 cm^3^ • FL volume: 167.1 cm^3^ • Branches perfused by the TL: BT, LCC, LSA, CT, SMA, LRA, IMA, RIIA, LIIA, LEIA • Branches perfused by the FL: RRA • Branches perfused by both the TL and the FL: REIA	• Type: AAA • Shape: fusiform • Max. diameter: ∼52.32 mm • Branches with dilation: BT, RIIA • Branches with stenosis: LCC, CT, LIIA	• Type: TAAD • Number of tears: 5 • PET area: ∼9.6 mm^2^ • TL volume: 163.7 cm^3^ • FL volume: 185.5 cm^3^ • Branches perfused by the TL: BT, LCC, LSA, CT, SMA, IMA, LEIA, LIIA • Branches perfused by both the TL and the FL: LRA, RRA, REIA, RIIA
Brachial systolic pressure (mmHg)	146	120	140
Brachial diastolic pressure (mmHg)	87	87	83
Heart rate (bpm)	87	63	82

TBAD, type-B aortic dissection; AAA, abdominal aortic aneurysm; TAAD, type-A aortic dissection; PET, primary entry tear; TL, true lumen; FL, false lumen; BT, brachiocephalic trunk; LCC, left common carotid; LSA, left subclavian artery; CT, celiac trunk; SMA, superior mesenteric artery; RRA, right renal artery; LRA, left renal artery; IMA, inferior mesenteric artery; REIA, right external iliac artery; RIIA, right internal iliac artery; LEIA, left external iliac artery; LIIA, left internal iliac artery.

The study protocol was approved by the institutional review board of Union Hospital, Tongji Medical College, Huazhong University of Science and Technology. Since the data involved in this study is retrospective, the requirement for informed consent was waived and anonymized data was used.

### 2.2 Clinical data

Computed tomography angiography (CTA) sequences of three patients were acquired using a 3rd generation Dual-Source CT scanner (SOMATOM Force; Siemens AG, Erlangen, Germany; 100 kV, 250 mA s, rotation time: 0.25 s, field of view (FOV): 250 mm, slice thickness: 0.5 mm, reconstruction kernel: Bv40, contrast agent: Iomeron 400; Bracco, Milan, Italy) with in-plane resolution of 0.5 mm and inter-slice distance of 0.5 mm for the reconstruction of the aorta, as shown in Supplementary Figure S1. Pre-treatment brachial blood pressure and heart rate were also measured and are summarized in Table 1.

### 2.3 Segmentation

The aorta geometries were reconstructed from CTA images using the image-processing software DetecModeling (Boea Wisdom, Hangzhou, China) (Ding et al., 2024). The reconstructed aorta, starting from the ascending aorta and extending to the internal and external iliac arteries, was cut and patched to form the inlet and outlet boundaries of the computational domain.

### 2.4 Geometry repair

To obtain a patient-specific healthy aortic model, the lesional aorta was manually repaired to generate a near-healthy reference geometry. To ensure the validity of the geometric repair, all modifications were independently performed by two experienced technicians under the guidance of a radiologist and a vascular surgeon. In cases of disagreement, a senior technician provided the final judgment.

**FIGURE 1:**
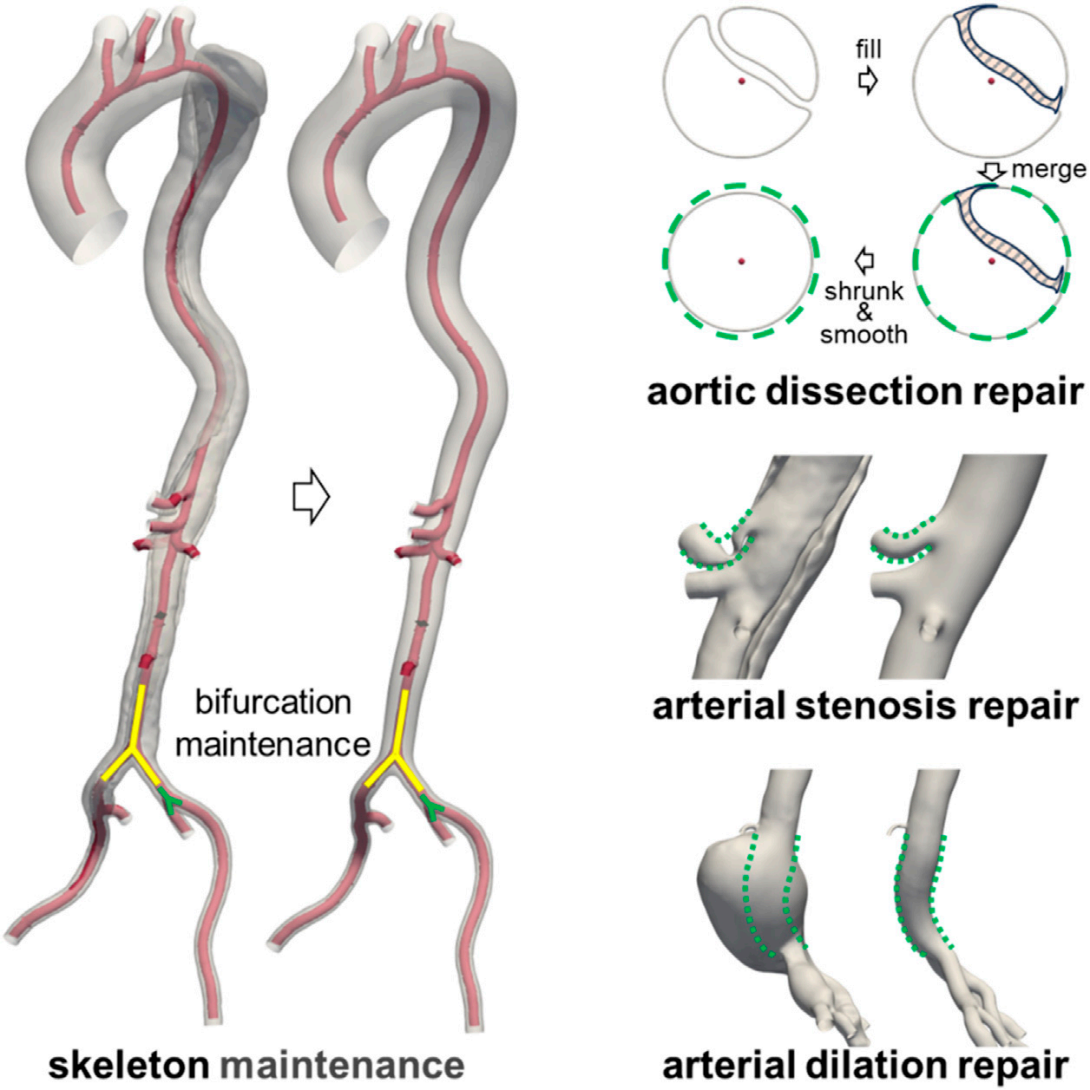
Manual repair of the diseased aortic model.

The repair process of the diseased aorta is illustrated in Figure 1. Since the CTA scans of the diseased aorta were obtained relatively soon after the onset of the disease, its overall structure remained largely unaffected (Li et al., 2023; Wen et al., 2023). Therefore, the original vascular framework was preserved during the repair process. Furthermore, as the arterial disease had minimal impact on the location and geometry of bifurcation points (Ford et al., 2009), their anatomical structures were retained in the reference model. For cases of aortic dissection, the intimal flap was first filled to merge the true and false lumens. Subsequently, the merged model underwent contraction and smoothing, as the presence of the intimal flap and blood perfusion in the false lumen could contribute to aortic expansion. In cases of abnormal stenosis or dilation, the vessel diameter was adjusted based on the diameter of unaffected segments within the same anatomical region. A comparison between the lesional geometry and the repaired near-healthy reference geometry is provided in Supplementary Figure S2.

### 2.5 Meshing

The spatial discretization of both the lesional and reference geometries was carried out using the mesh tool integrated in DetecFluid (Boea Wisdom, Hangzhou, China). Given the complex morphology of the aorta, tetrahedral elements were employed for meshing. To ensure grid independence, three different grid resolutions were tested (see Supplementary Material), and a suitable configuration was selected for each aortic model. In the final meshes, the number of cell elements ranged from approximately 2.5–4.4 million across all patient-specific models, providing sufficiently fine resolution to capture the hemodynamic features. Detailed mesh statistics are provided in Supplementary Material.

### 2.6 Estimation of flow division in aortic branches

**FIGURE 2:**
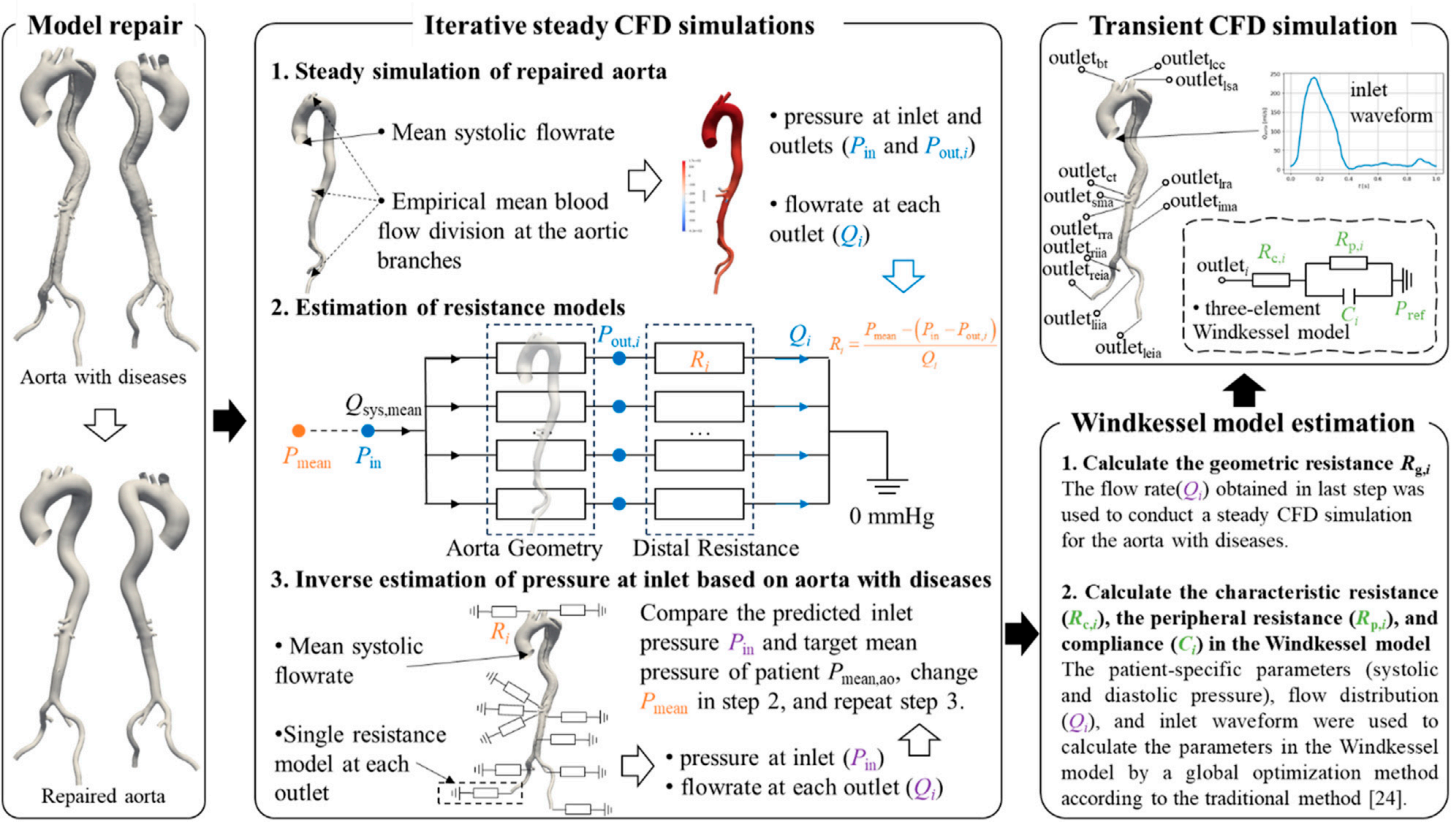
Iterative framework for estimating the flow division in branches of the diseased aorta.

As illustrated in Figure 2, an iterative framework based on steady-state CFD simulations was developed to estimate the flow division in the downstream branches of diseased aortic models. Steady-state simulations were first conducted on the repaired reference geometry to obtain inlet and outlets pressures. The outlet pressures were subsequently converted into distal resistance models and prescribed at the outlets of the diseased geometry. Subsequently, steady-state simulations were performed on the diseased model to predict the inlet pressure, which was compared against the target value. Based on this discrepancy, the outlet resistances were updated, and the simulations were repeated until convergence was achieved. The boundary conditions employed in each simulation are described in detail below.

In the steady-state CFD simulation of the reference model, the mean systolic flow rate, derived from empirical flow waveforms presented in Figure 2, was prescribed at the inlet, as this approach has been shown to effective predict mean blood pressure and vascular resistance (Li and Mao, 2023). The downstream flow division was assigned based on widely used measurements from previous studies (Je and Dn, 1994; Moore and Ku, 1994). Specifically, 30% of the cardiac output was allocated to the supra-aortic branches, with further distribution among them proportional to their cross-sectional areas. The celiac trunk received 15.5% of the cardiac output, while the superior mesenteric artery, the left renal artery, and the right renal artery each accounted for 10.5%. If the inferior mesenteric artery was present, it received 0.5% of the cardiac output. The remaining flow was equally divided between the left and right iliac arteries, with 30% directed into the internal iliac artery and 70% into the external iliac artery. Although these flow division values are based on imprecise historical measurements, they remain widely accepted in hemodynamic studies (Bonfanti et al., 2017; Bonfanti et al., 2019). Importantly, this does not affect the validation of the proposed method. More accurate flow division data from healthy individuals could be incorporated into future studies as they become available.

Based on the inlet pressure, outlet pressures, and flow rates predicted by the steady-state CFD simulation of the reference model, along with the empirical mean blood pressure values, the resistance at each outlet in the resistance model can be calculated using Equation 1:

$$R_{i}=\frac{P_{\text{mean}}-\left(P_{\text{in}}-P_{\text{out},i}\right)}{Q_{i}}$$ (1)

Where $R_{i}$ represents the resistance of the *i*th branch artery, mmHg·s·ml^-1^; $P_{\text{mean}}$ is the empirical mean blood pressure, which will be adjusted according to the discrepancy between the predicted inlet pressure and the target inlet pressure of the diseased aorta; $P_{\text{in}}$ is the predicted inlet pressure of the reference model, mmHg; $P_{\text{out},i}$ is the predicted pressure at the *i*th branch outlet of the reference model, mmHg; and $Q_{i}$ is the flow rate at the *i*th outlet of the reference model, ml·s^-1^.

Next, a steady-state CFD simulation was conducted on the diseased aorta. The inlet boundary condition was set as a mass flow rate equal to that of the reference model. At the outlets, the resistance model was imposed, allowing the outlet pressure to vary dynamically with the flow rate. This simulation provided a predicted inlet pressure for the diseased aorta, which was then compared to the target value. Given that only brachial blood pressure was available for each patient, an empirical formula was used to estimate the mean pressure at the aortic inlet, as presented in Equations 2–4 (Stokes et al., 2021):

$$P_{\text{mean},\text{ao}}=0.4P_{\text{sys},\text{ao}}+0.6P_{\text{dia},\text{ao}}$$ (2)

$$P_{\text{sys},\text{ao}}=0.83P_{\text{sys},\text{bra}}+0.15P_{\text{dia},\text{bra}}$$ (3)

$$P_{\text{dia},\text{ao}}=P_{\text{dia},\text{bra}}$$ (4)

Where $P_{\text{mean},\text{ao}}$ is the mean pressure at the aortic inlet, mmHg; $P_{\text{sys},\text{ao}}$ is the aortic systolic pressure, mmHg; $P_{\text{dia},\text{ao}}$ is the aortic diastolic pressure, mmHg; $P_{\text{sys},\text{bra}}$ is the brachial systolic pressure, mmHg; and $P_{\text{dia},\text{bra}}$ is the brachial diastolic pressure, mmHg.

The threshold for the discrepancy between the predicted and target inlet pressures was set at 1%. If the difference exceeded this threshold, the empirical mean pressure in Equation 1 was adjusted, and the steady-state CFD simulation of the diseased model was repeated. The iteration continued until discrepancy fell below 1%, at which point convergence was achieved. At this stage, the outlet resistance model was deemed representative of the patient’s physiological condition, and the predicted flow division in the aortic branches was considered a more patient-specific estimation.

### 2.7 CFD simulation for hemodynamic analysis

Transient CFD simulations were performed for each diseased aorta to analyze its hemodynamic characteristics. The inlet boundary condition was prescribed using a flow rate waveform (Figure 2) from the literature (Gallo et al., 2016), scaled according to patient-specific physiological data, and imposed with a plug flow profile. At the outlets, the three-element Windkessel was implemented, with its parameters estimated using the fast approach proposed by Li and Mao (Li and Mao, 2023). In the estimation process, the previously predicted patient-specific flow division in the aortic branches was used.

Additionally, to compare and assess the performance of the proposed method, a traditional scheme based on empirical flow division from healthy individuals (Bonfanti et al., 2017; 2019), specifically, the outlet flow rate used in step 1 in Figure 2, was also employed for Windkessel parameter estimation. In the following text, the results obtained using the previously predicted flow rate are labeled as the “proposed method”, while the results from the traditional scheme are labeled as the “traditional method”.

All CFD simulations were conducted by the software DetecFluid (Boea Wisdom, Hangzhou, China). It is a highly efficient GPU-based software for solving the equations of continuity and momentum conservation using a finite-volume methodology (Liu et al., 2025). Blood was assumed to be an incompressible Newtonian fluid with a density of 1,060 kg m^-3^ and a dynamic viscosity of 0.0035 Pa s. The vessel wall was defined as rigid with a no-slip boundary condition. Given the relatively high blood velocity and potential turbulence in the aorta, the standard *k* - *ε *model (Moukalled et al., 2016) was employed due to its numerical stability and its demonstrated applicability in aortic hemodynamics (Long Ko et al., 2017; Etli et al., 2021; Hohri et al., 2021). The standard wall function was used to resolve the viscous sublayer. A second-order scheme was used for spatial discretization and a first-order implicit scheme for temporal integration. The SIMPLE algorithm (Moukalled et al., 2016) was used to couple pressure and velocity equations. The converged residuals for all variables were 10^–4^. Additionally, for the transient CFD simulation, the cardiac cycle was divided into 1,000 time-steps. The transient simulation was performed over five cardiac cycles, and the analysis was conducted based on the data from the final cycle.

### 2.8 Flow analysis

Several metrics related to blood pressure, flow patterns, and wall shear stress were used to characterize the aortic hemodynamics. For temporal statistical metrics, every tenth timestep from the final cycle was used.

#### 2.8.1 Blood pressure

Intravascular pressure is believed to play a significant role in the progression of cardiovascular diseases. For instance, elevated static pressure has been linked to the formation and rupture of aneurysms (Suzuki et al., 2016; Jiang et al., 2019; Huang et al., 2020; Yi et al., 2023). In this study, the distribution of static pressure was analyzed across ten sections of the aortic trunk. Additionally, the transmural pressure (TMP = *P*_TL_–*P*_FL_, where *P*_TL_ and *P*_FL_ are the pressure in the TL and FL, respectively) across the intimal flap was calculated and analyzed for Patients 1 and 3.

#### 2.8.2 Flow pattern

The flow divisions at branch outlets were calculated and compared. Moreover, the velocity and helicity distributions across the same ten sections were illustrated. The helicity was quantified using the local normalized helicity (LNH) (Gallo et al., 2012), as shown in Equation 5.

$$\text{LNH}=\frac{v\cdot \left(\nabla \times v\right)}{\left|v\right|\left|\nabla \times v\right|}$$ (5)

Where v is the velocity vector, m·s^−1^.

#### 2.8.3 Wall shear stress (WSS) related metrics

WSS-related metrics have been shown to be associated with the progression of aortic diseases (Boyd et al., 2016; Xu et al., 2018). Four commonly used metrics including time averaged WSS (TAWSS), oscillatory shear index (OSI), relative residence time (RRT), and aneurysm formation indicator (AFI) were selected and calculated by Equations 6–9.

$$\text{TAWSS}=\frac{1}{T}\int_{0}^{T}\left|\tau_{w}\right|dt$$ (6)

$$\text{OSI}=\frac{1}{2}\left(1-\frac{\left|\int_{0}^{T}\tau_{w}dt\right|}{\int_{0}^{T}\left|\tau_{w}\right|dt}\right)$$ (7)

$$\text{RRT}=\frac{1}{\left(1-2\times \text{OSI}\right)\times \text{TAWSS}}=\frac{1}{\frac{1}{T}\left|\int_{0}^{T}\tau_{w}dt\right|}$$ (8)

$$\text{AFI}=\frac{\tau_{w}\cdot \int_{0}^{T}\tau_{w}dt}{\left|\tau_{w}\right|\left|\int_{0}^{T}\tau_{w}dt\right|}$$ (9)

Where *T* is the cardiac cycle, $\tau_{w}$ is the WSS vector. TAWSS, OSI, RRT were calculated based on the data from the whole final cycle, while AFI was calculated at the mid-systolic deceleration (Mantha et al., 2006).

## 3 Results

### 3.1 Flow division in aortic branches

**FIGURE 3:**
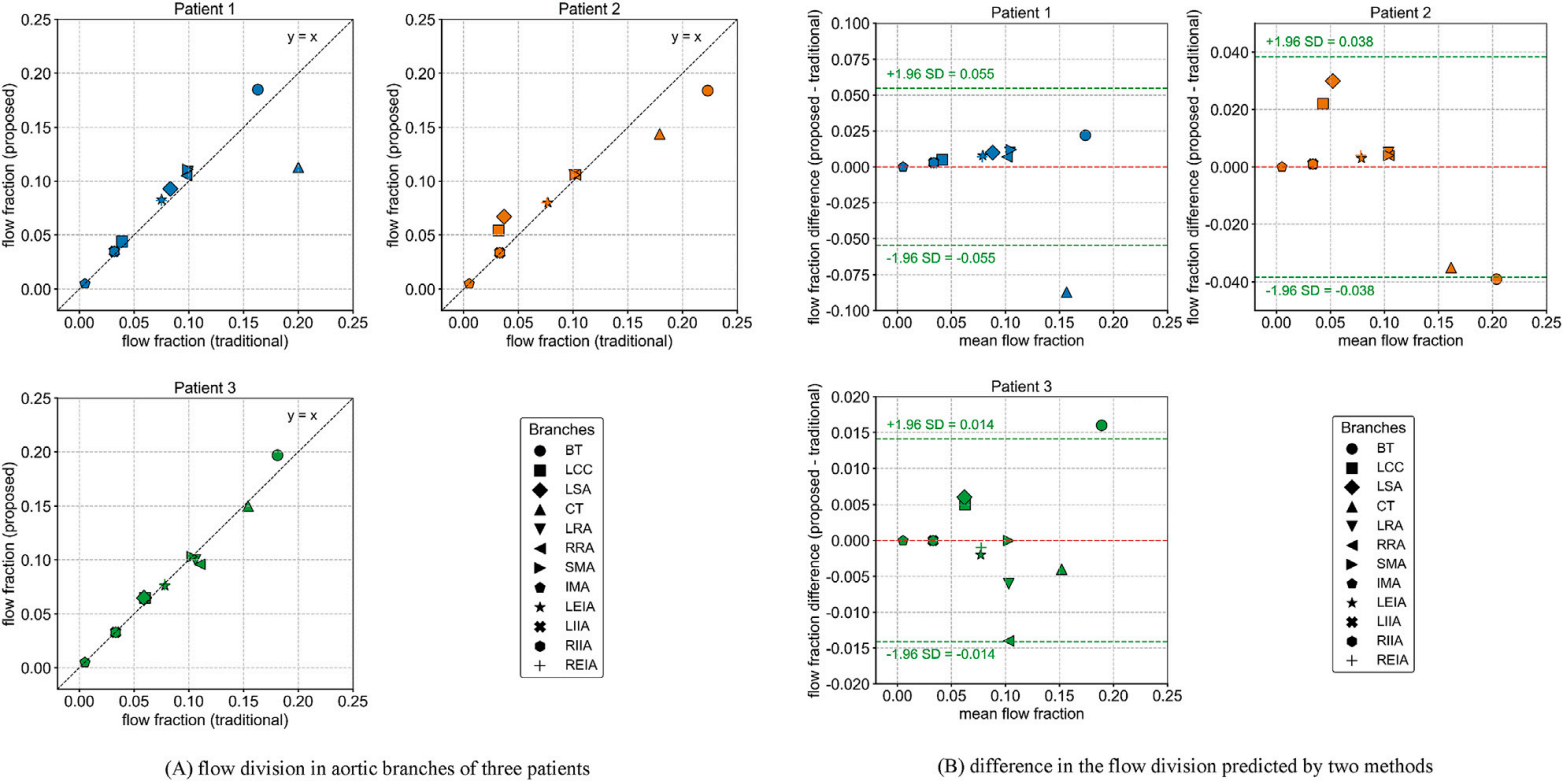
Flow division in aortic branches predicted by the traditional and proposed methods. **(A)** flow division in aortic branches of three patients **(B)** difference in the flow division predicted by two methods.

Figure 3 illustrates the flow division in aortic branches predicted by the traditional and proposed methods. The specific flow rate values for each branch are provided in Supplementary Table S3. A strong correlation was observed between the two methods in predicting aortic branch flow division (Patient 1: *r* = 0.865, Patient 2: *r* = 0.973, Patient 3: *r* = 0.991). However, noticeable differences were found in certain branches, particularly those with severe stenosis, such as the celiac trunk in Patients 1 and 2. The proposed method predicted lower flow rates in these arteries, which is considered more physiologically realistic, resembling the “steal” phenomenon observed in patients with artery occlusions that redistribute blood flow to meet metabolic demands (Yamashiro et al., 2002; Hatzidakis et al., 2015). The Bland-Altman plot further indicates that the differences in predicted flow fractions for some branches approach or exceed the 95% limits of agreement.

### 3.2 Estimated parameters of the windkessel models

**FIGURE 4:**
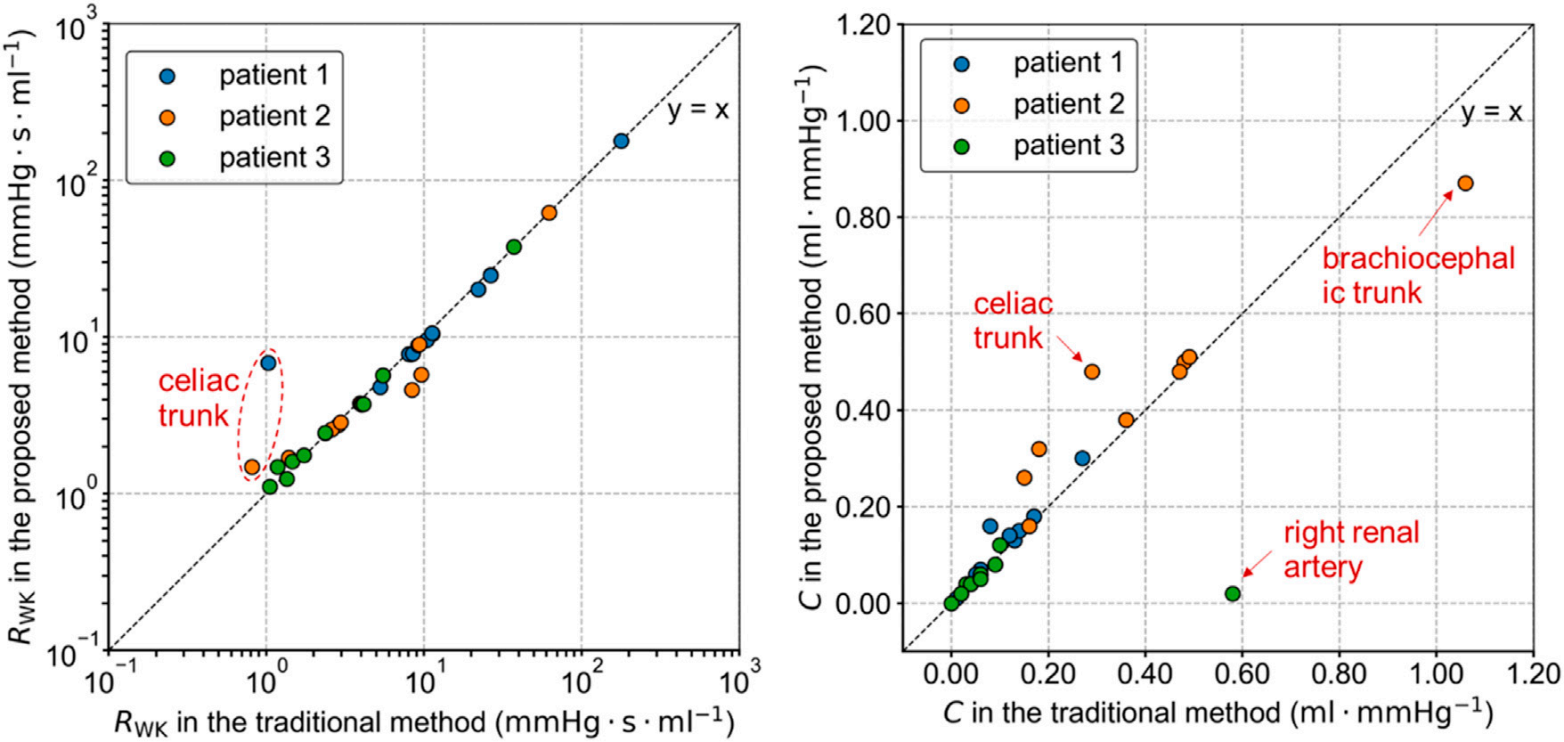
Parameters of the Windkessel model estimated by the traditional and proposed methods.

The total resistance *R*_WK_ and compliance *C* of the Windkessel models estimated by the traditional and proposed methods differed, as shown in Figure 4. These variations in parameters are related to the severity of the lesions and the differences in target flow rates in each branch. In the proposed method, the flow rate in the stenotic branches decreased and was redistributed to other branches. As a result, the total resistance of most Windkessel models in Patients 1 and 2 decreased, while the resistance of severely stenotic branches, such as the celiac trunk, increased. The variation in capacitance values of the Windkessel model was more complex, as it was influenced by both the pressure changes due to flow rate variations and volume changes associated with vascular lesions. Among all branches, the greatest change in capacitance occurred in the right renal artery of Patient 3, where it decreased from 0.577 mL mmHg^-1^ in the traditional method to 0.022 mL mmHg^-1^ in the proposed method.

### 3.3 Blood pressure

**FIGURE 5:**
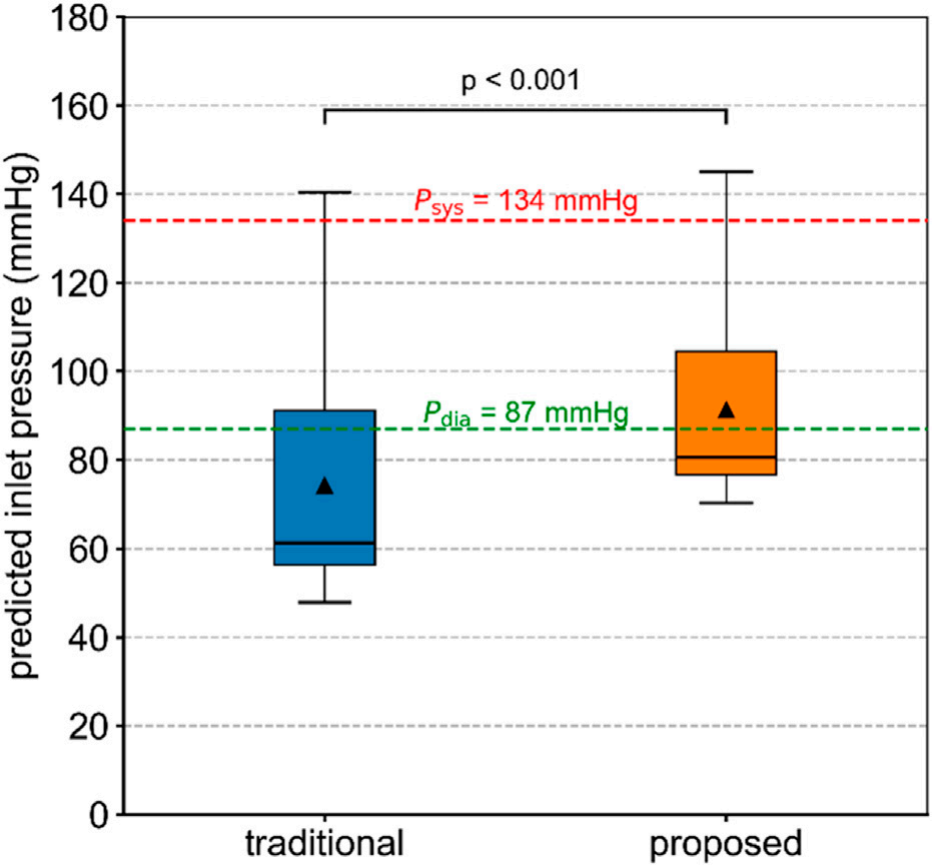
Inlet pressure of the diseased aorta in Patient 1, predicted by the traditional and proposed methods. The Wilcoxon signed-rank test was used to compare the difference.

The inlet pressures of the diseased aorta in Patient 1, predicted by the traditional and proposed methods, are compared and shown in Figure 5. The predicted inlet pressures differed from the measured aortic systolic and diastolic pressures. The proposed method performed better in reproducing the inlet blood pressure. In contrast, the traditional method assigned flow rates typical of healthy conditions to the stenotic branches, resulting in non-physiological pressure losses that influenced the simulation outcomes. The predicted systolic and diastolic pressures for Patients 2 and 3 are summarized in Supplementary Table S3. For Patient 3, the predicted inlet pressure from both methods significantly differed from the actual values. This discrepancy may be related to the limitations of the tuning strategy used in Windkessel models (Li and Mao, 2023).

**FIGURE 6:**
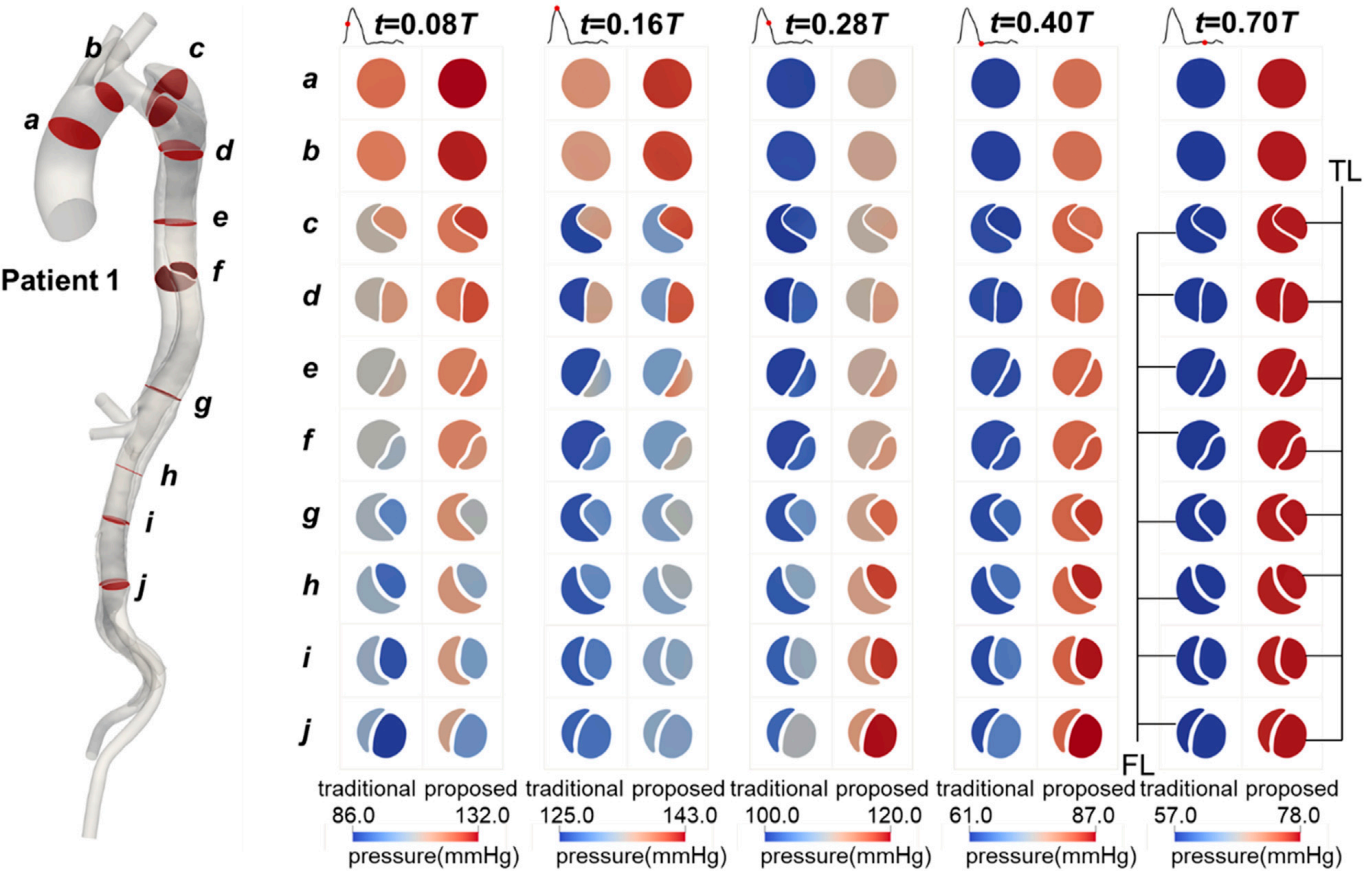
Contours of blood pressure on the selected planes in Patient 1 at mid-systolic acceleration, peak systole, mid-systolic deceleration, early diastole, and mid-diastole.

The blood pressure distributions on the selected planes in Patient 1 at five time points are shown in Figure 6. The values predicted by the proposed method were higher than those predicted by the traditional method, and the distribution patterns were similar. Additionally, the blood pressure variations over time during the cardiac cycle were comparable. Similar trends were observed in Patients 2 and 3, as shown in Supplementary Figures S3,S4.

**FIGURE 7:**
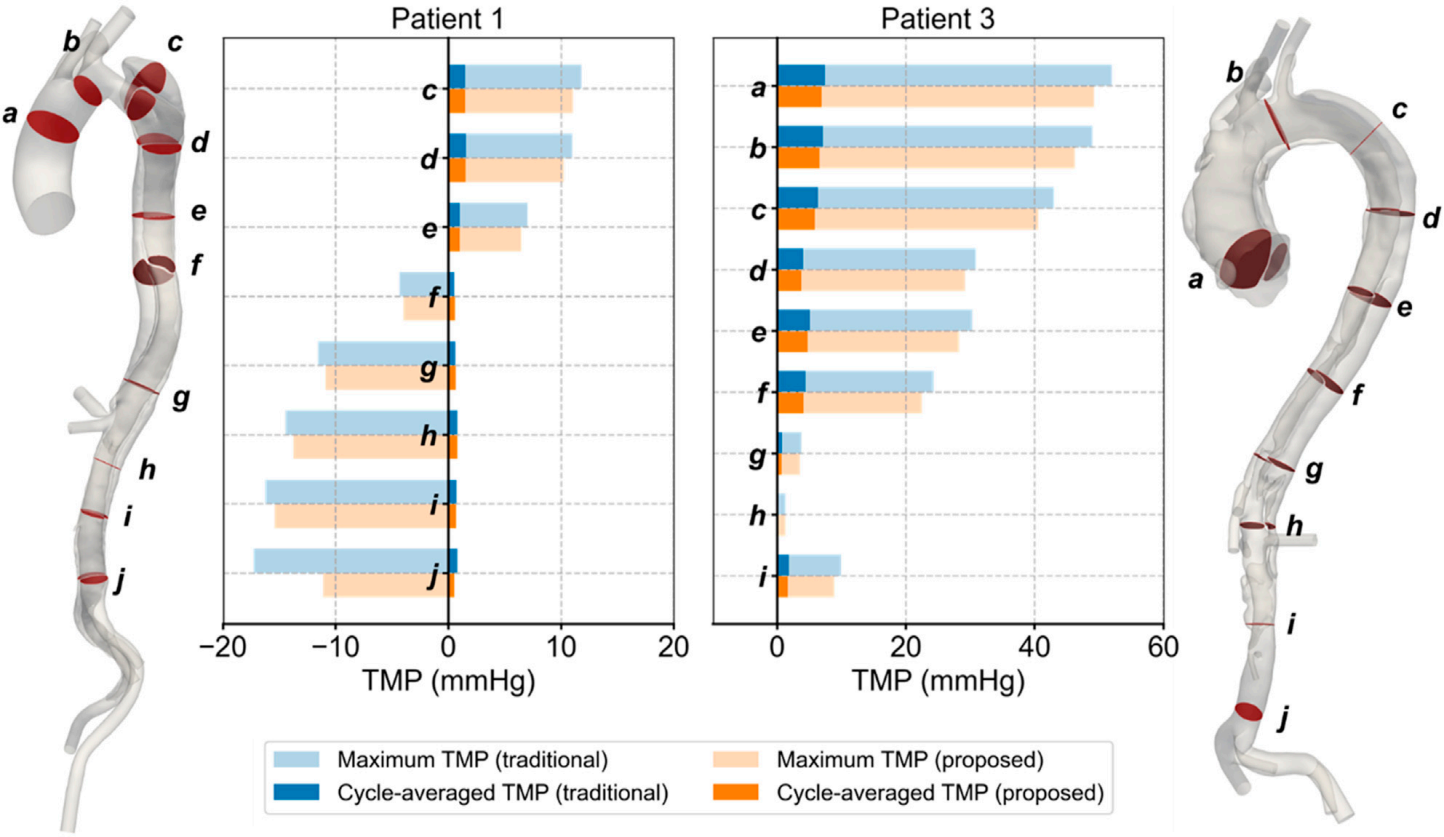
Spatial and temporal distributions of transmural pressure on the selected planes in Patients 1 and 3.

The TMP values for Patients 1 and 3, calculated by both methods, are shown in Figure 7. The values predicted by the proposed method differed from those predicted by the traditional method, with the traditional method generally overestimating the absolute TMP values. The difference was more pronounced and significant (p < 0.01) in Patient 3, primarily due to two severe stenoses in the true lumen of the descending aorta, located around planes *d* and *g*. These stenoses had a broader impact, leading to the observed discrepancies. Both methods predicted the same balance points where TMP equaled zero: for Patient 1, this balance point was between planes *e* and *f*, and for Patient 3, it was near plane *h*. The maximum difference in TMP values predicted by the two methods exceeded 5 mmHg, occurring near the iliac bifurcation in Patient 1, around plane *j*.

### 3.4 Flow patterns

**FIGURE 8:**
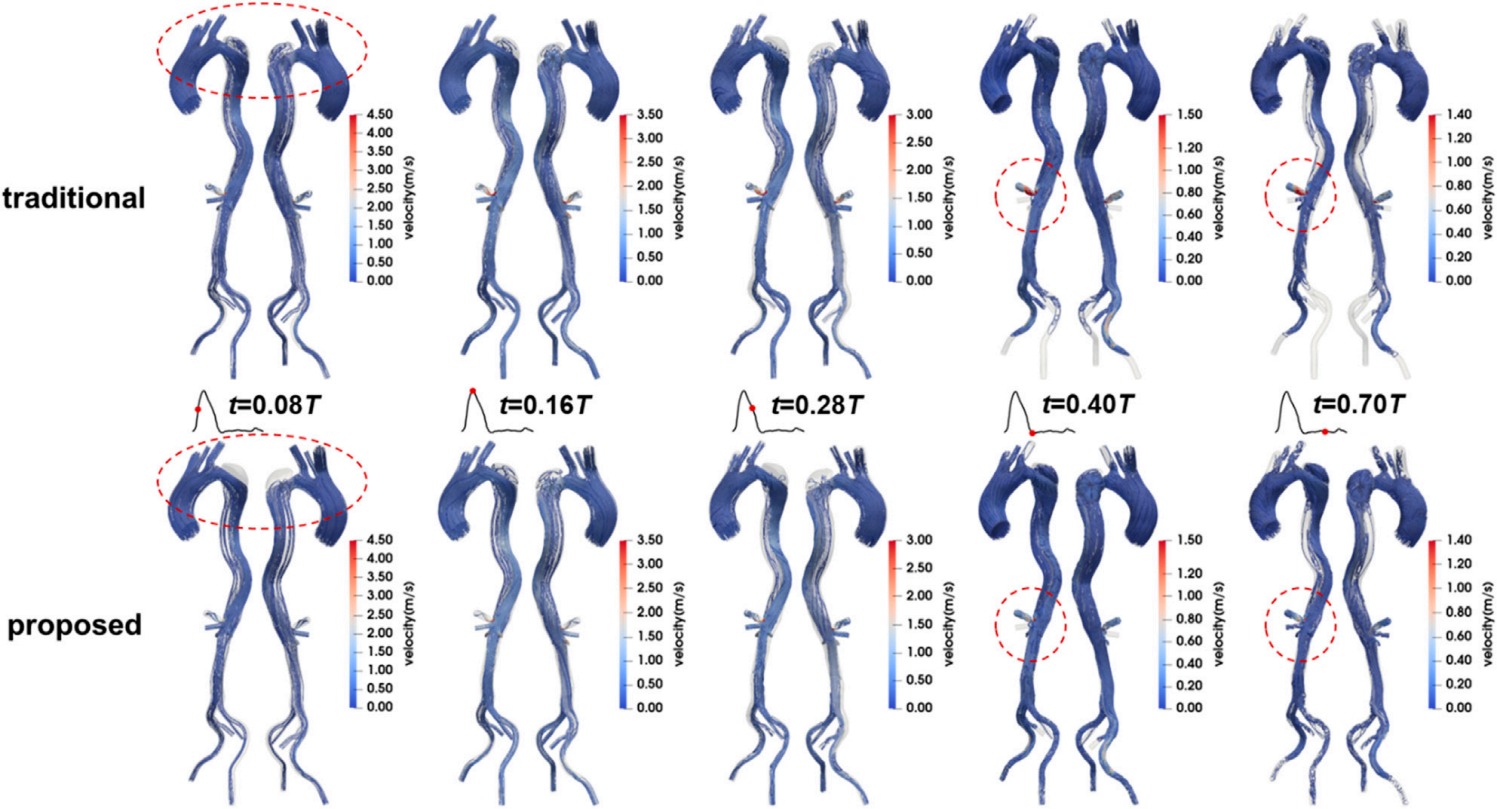
Velocity streamlines in Patient 1 at mid-systolic acceleration, peak systole, mid-systolic deceleration, early diastole, and mid-diastole.

**FIGURE 9:**
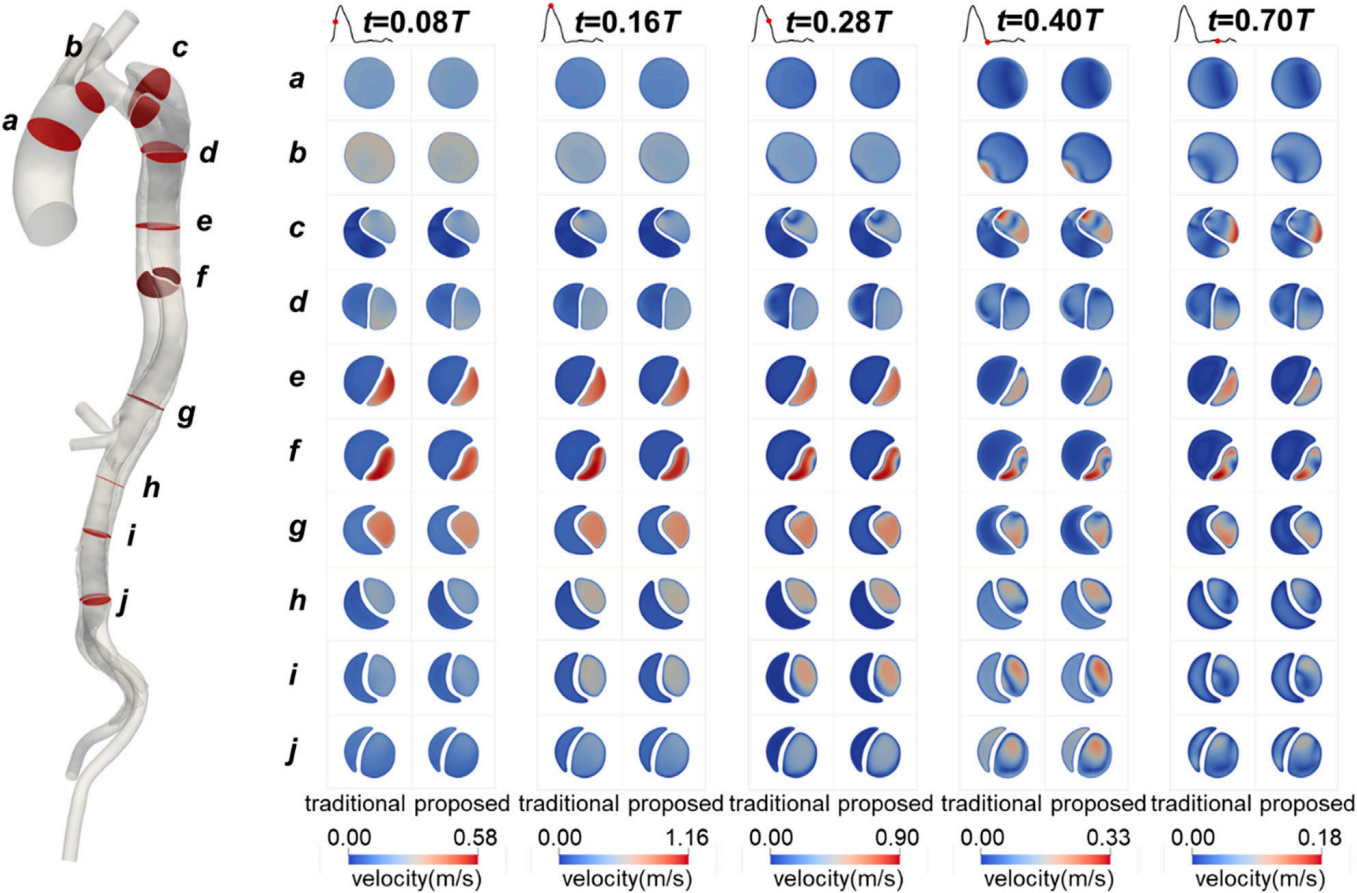
Contours of velocity on the selected planes in Patient 1 at mid-systolic acceleration, peak systole, mid-systolic deceleration, early diastole, and mid-diastole.

The flow patterns in the aorta of Patient 1 are presented here. Results for Patients 2 and 3 can also be found in the Supplementary Material. Figure 8 illustrates the flow patterns in Patient 1 at five representative time points, and Figure 9 shows the velocity distribution on the selected planes. Unlike the blood pressure distributions, the velocity distributions predicted by the two methods were similar. Aorta tears cause a significant reduction in the cross-sectional area of the TL, but most of the blood still flows into the TL (86.5% in the proposed method and 88.4% in the traditional method), resulting in a high blood velocity in the TL. For branches supplied by both TL and FL, there was a slight but not significant change in the proportion of source. In the REIA, the proportion of blood flow from the TL increased from 85.4% in the traditional method to 87.4% in the proposed method.

**FIGURE 10:**
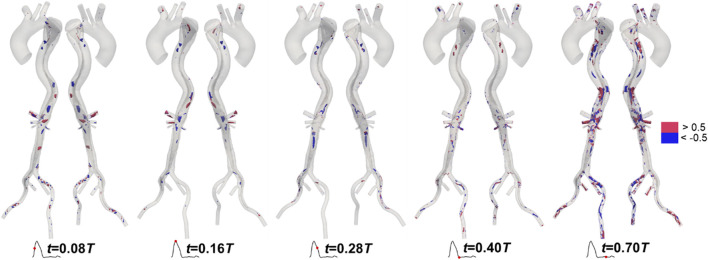
Differences in local normalized helicity in Patient 1 at mid-systolic acceleration, peak systole, mid-systolic deceleration, early diastole, and mid-diastole.

Like velocity, the distributions of LNH predicted by the two methods are similar. To maintain conciseness, these distributions are not shown here and can be found in the Supplementary Material. We calculated the difference in LNH by subtracting the values predicted by the traditional method from those predicted by the proposed method, as shown in Figure 10. The differences in LNH were scattered sporadically throughout the aorta, with more pronounced variations observed in the FL, which has a relatively large volume. From a temporal perspective, the differences were more pronounced during mid-systolic acceleration and the diastole phase compared to other times. This may be linked to the strong transition flow occurring between systole and diastole (Liu et al., 2012; Poelma et al., 2015).

### 3.5 WSS-related hemodynamics

**FIGURE 11:**
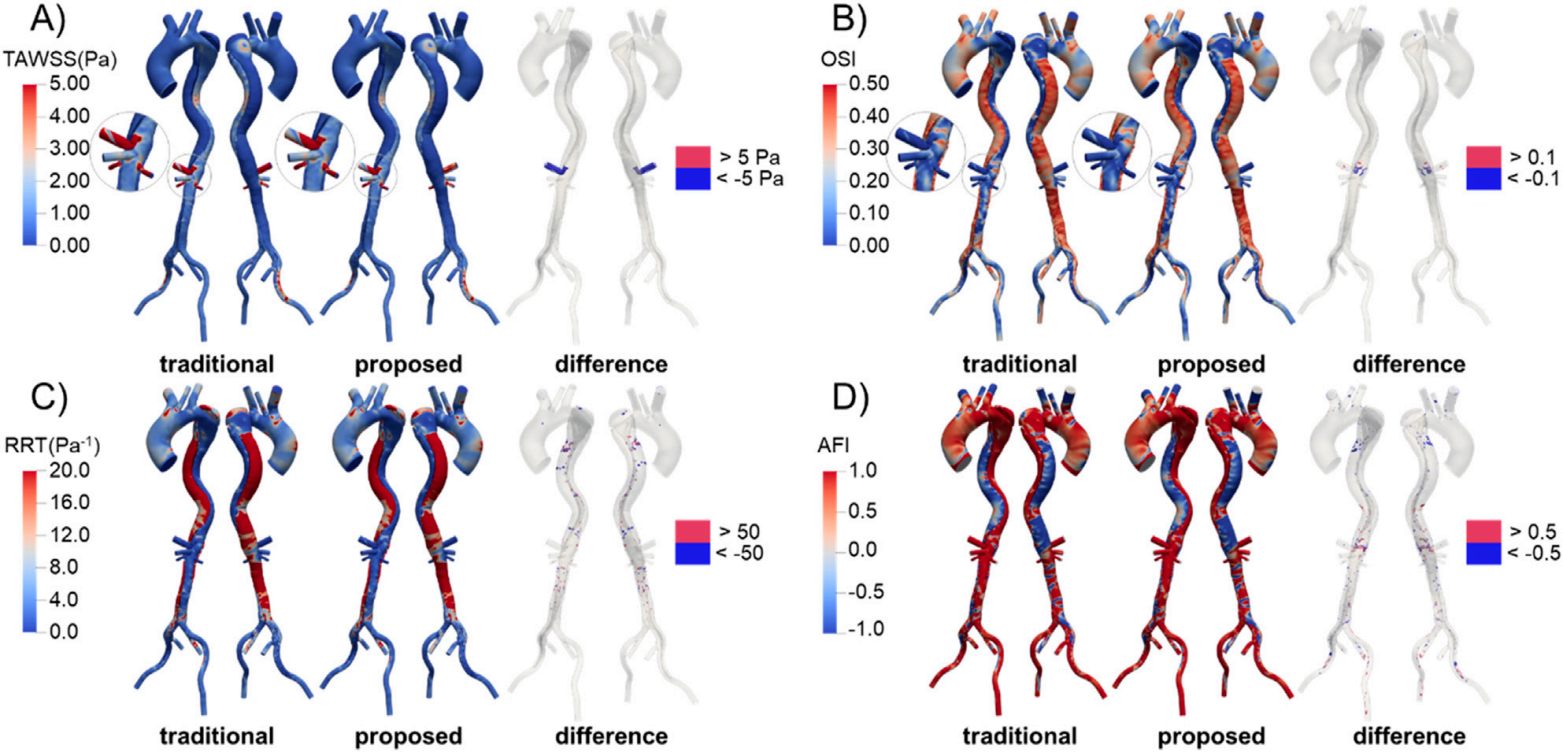
Distribution and difference of WSS-related metrics in Patient 1 predicted by the traditional and proposed methods. **(A)** TAWSS; **(B)** OSI; **(C)** RRT; **(D)** AFI.

The distributions of WSS-related metrics in Patient 1 are shown in Figure 11, with those for Patients 2 and 3 available in the Supplementary Material. The distributions predicted by the two methods were similar, especially for the metrics based on cycle-averaged data such as TAWSS, OSI, and RRT. This is understandable, as these indicators are derived from wall shear stress, which is determined by the velocity gradient near the vessel wall. As previously mentioned, the velocity distributions along the aorta trunk predicted by the two methods were comparable. In the TL, high blood velocity resulted in high and unidirectional WSS, characterized by higher TAWSS, lower OSI and RRT, and more uniform AFI. In contrast, the FL exhibited low and oscillating WSS, with lower TAWSS, higher OSI and RRT, and more varied AFI, due to the larger volume and lower flow rate.

We calculated the difference in these metrics by subtracting the values predicted by the traditional method from those predicted by the proposed method, as shown in Figure 11. In the celiac trunk, the TAWSS predicted by the proposed method was relatively lower due to the reduced flow rate. Differences in OSI were observed in areas near the visceral branches, where flow patterns were complex, while differences in RRT were scattered sporadically throughout the FL. For AFI, significant differences were widely distributed throughout the aorta, especially in the FL.

## 4 Discussion

### 4.1 Comparison with zero-pressure simulations

More realistic predictions of blood flow distribution enable more precise identification of malperfusion syndrome in patients. This represents a significant advancement over past studies that used simulations with zero-pressure outlets to assess the risk of malperfusion. Cheng et al. (2013) utilized transient simulations with zero-pressure outlets to analyze the hemodynamic characteristics of four patients with acute TBAD, two of whom developed complications of malperfusion. They suggested that higher flow rate into the FL might be related to malperfusion syndrome. However, their models did not include downstream branch arteries. Furthermore, a high flow rate into the FL does not necessarily result in malperfusion, as a patent FL can still supply blood to the distal arteries (Bonfanti et al., 2019). Alimohammadi et al. (2014) developed a patient-specific simulation tool for aortic dissection, in which the downstream flow division was first determined using a steady-state CFD simulation with zero-pressure outlets to tune the Windkessel model parameters for multi-scale simulations. Their results showed that 39.4% of the inlet flow went through the BT, leaving only 45.0% for the descending aorta, which contributed to lower limb and/or vital organ malperfusion. However, simulations using zero-pressure outlets likely overestimate the risk of malperfusion in downstream branches because the flow division was directly determined by the geometric resistance in the reconstructed aorta model. For comparison, we conducted simulations with zero-pressure outlets for Patient 1, following the methodologies of Cheng et al. (2013) and Alimohammadi et al. (2014). Table 2 illustrates the flow rate in branches predicted by these simulations. The “0Pa_Windkessel_target” values represent the flow division predicted by the steady-state simulation with zero-pressure outlets. The “0Pa_Windkessel” values denote the flow rates predicted by the multi-scale simulation with Windkessel models, whose parameters were estimated using the flow division in “0Pa_Windkessel_target”. The “0Pa_Transient” values show the flow division predicted by the transient simulation with zero-pressure outlets. It is evident that the flow distributions predicted by these simulations are similar, with approximately 48% of the inlet flow passing through the BT, similar to Alimohammadi et al.‘s study (Alimohammadi et al., 2014). The flow rates in the three branches on the arch were much higher than those in distal branches, which is clearly inconsistent with the physiological condition.

**TABLE 2:** Flow division predicted by simulations with zero-pressure outlets.

Location	Zero-pressure outlets		
	0Pa_Transient	0Pa_Windkessel	0Pa_Windkessel_target
BT	49.1%	46.6%	47.0%
LCC	6.0%	10.8%	10.9%
LSA	20.8%	23.5%	23.7%
CT	0.3%	0.4%	0.4%
LRA	2.2%	1.8%	1.8%
RRA	0.8%	1.7%	1.7%
SMA	6.9%	5.1%	5.1%
IMA	0.2%	0.3%	0.3%
LEIA	3.1%	2.4%	2.2%
LIIA	4.3%	2.9%	2.7%
RIIA	3.7%	2.5%	2.3%
REIA	2.6%	1.9%	1.9%

Furthermore, more accurate predictions of blood pressure and WSS-related metrics allow for a more precise evaluation of aortic diseases progression risks, including the obstruction of the TL (Bonfanti et al., 2019; Xu et al., 2021), the evolution of the FL (Chen et al., 2013; Xu et al., 2018), the dilation and rupture of aneurysms (Teng et al., 2022), and the formation of luminal thrombosis (Naim et al., 2016).

### 4.2 Impact of manual model repair

As introduced before, the proposed method involves manual repair of the diseased aorta model, which can introduce additional errors. Given that the level of smoothing in model reconstruction significantly impacts hemodynamic predictions (Paritala et al., 2023), we conducted a preliminary investigation into the effect of the smoothing factor on the results. Specifically, the repaired aorta of Patient 1 was smoothed to varying degrees, and the flow division in branches was recalculated using the proposed method. The results, presented in Table 3, indicate that smoothed models yielded more consistent flow rates with the proposed method and deviated significantly from the flow division predicted by the traditional method. Future work should focus on developing automated model repair techniques to strengthen the robustness of the proposed method. Ford et al. (2009) developed an objective and automated technique for digitally repairing arteries with saccular aneurysmal lesions. However, for more complex aortic conditions, such as fusiform aneurysms or aortic dissection, efficient automated repair algorithms remain unavailable.

**TABLE 3:** Target flow rates predicted by steady-state simulations for various repaired geometries.

Location	Smoothing degree			Proposed method	Traditional method
	Mild	Moderate	Severe		
BT	19.4%	19.4%	19.4%	18.9%	17.2%
LCC	4.4%	4.3%	4.3%	4.5%	4.1%
LSA	9.1%	9.0%	8.9%	9.5%	8.7%
CT	9.2%	9.2%	9.1%	9.1%	15.5%
LRA	11.2%	11.3%	11.4%	11.2%	10.5%
RRA	10.9%	11.0%	11.2%	10.8%	10.5%
SMA	11.3%	11.3%	11.3%	11.4%	10.5%
IMA	0.5%	0.5%	0.5%	0.5%	0.5%
LEIA	8.4%	8.4%	8.4%	8.5%	7.9%
LIIA	3.6%	3.6%	3.6%	3.6%	3.4%
RIIA	3.6%	3.6%	3.6%	3.6%	3.4%
REIA	8.4%	8.4%	8.3%	8.4%	7.9%

### 4.3 Limitations and future perspectives

There are several limitations in this study. First, while we showed that the proposed method could reproduce inlet blood pressure closer to the physiological data of patients, some persistent differences remained. These discrepancies arose because the Windkessel model parameters were estimated by a steady-state simulation with mean systolic blood flowrate at the inlet. This strategy is more effective for healthy aortas, where geometric resistances of branches vary similarly under different inflow rates. However, in stenotic branches of diseased aortas, the variation in resistance with inflow rate differs significantly from that of vessels with normal diameters, leading to less satisfactory predictions. In Li and Mao’s study (Li and Mao, 2023), the difference between the reproduced inlet pressure and physiological data also increased after the artificial narrowing of a branch. In addition, estimating Windkessel parameters from steady-state simulations has an inherent drawback: arterial compliance is neglected. As a result, the predicted inlet pressure ( $P_{\text{in}}$ ) in Equation 1 is overestimated, leading to an underestimated resistance term ( $R_{i}$ ). When these parameters are subsequently applied in transient simulations, the Windkessel model tends to underestimate the mean arterial pressure across the cardiac cycle. Fortunately, the framework proposed in this study is adaptable to any Windkessel model tuning strategy that requires target flow rates, including those based on 0D simulations (Stokes et al., 2021). In the future, efforts should focus on improving strategies to better reproduce patient physiological data.

In addition, several common assumptions and simplifications in CFD simulations, such as the rigid vessel wall, empirical inflow waveform combined with a plug flow profile, and Reynolds-averaged turbulence models, may reduce accuracy. Previous studies have shown that assuming a rigid wall tends to overestimate flow velocity and wall shear stress (Ene et al., 2014; Bonfanti et al., 2018). Compared with healthy aorta, neglecting wall compliance in diseased aortas with local stenoses, as considered in this study, may introduce larger errors. On the one hand, wall deformation can substantially alter the local resistance at the stenotic site and thereby affect flow distribution; on the other hand, in some pathologies, such as aortic dissection, intimal flap motion may cause dynamic obstruction (Kim et al., 2023), leading to significant changes in hemodynamics. Simplifications in the inlet boundary condition, particularly the use of a plug profile, have also been shown to compromise the accuracy of wall hemodynamic predictions in the ascending aorta (Stokes et al., 2023a). Moreover, the Reynolds-averaged turbulence model adopted in this study (the standard k - ε model) may not adequately capture the transition between laminar and turbulent flow. Performing large-eddy simulations with finer meshes would enable the resolution of smaller-scale vortical structures and yield more accurate wall hemodynamic characteristics (Manchester et al., 2022; Cheng et al., 2025). Nevertheless, while these assumptions and simplifications may limit the absolute accuracy of the simulations, they do not undermine the demonstration that the proposed approach outperforms the traditional one.

Finally, the actual flow rate in a patient’s aortic branches is also regulated by the sympathetic nervous and endocrine systems (Leito-Rocha et al., 2012), which were not explicitly considered in this study. The sympathetic nervous system is known to regulate blood pressure by controlling peripheral resistance and cardiac output (Luo, 2003). In this study, different inlet pressures were assigned to the repaired and diseased aorta, implying that the effect of the sympathetic nervous system was, to some extent, implicitly considered. If physiological parameters such as blood pressure and cardiac output in the healthy state were available, the proposed method could be further refined. Additionally, future studies should validate the accuracy of the proposed method using clinically measured flow data, for example, obtained by four-dimensional flow magnetic resonance imaging (4D-Flow MRI), two-dimensional phase-contrast magnetic resonance imaging (2D PC-MRI), or Doppler ultrasound.

## 5 Conclusion

In summary, a novel framework has been developed to determine the flow division in aortic branches of patients with aortic diseases. This framework includes manual repair of diseased geometry and iterative steady-state CFD simulations to predict the target flow division for the lesional model. The predicted flow division is then used to estimate Windkessel model parameters for multi-scale simulations.

Applications in three patients demonstrated that the proposed method predicted a significant reduction in blood flow in stenotic branches, aligning more closely with physiological expectations. Furthermore, the method improved accuracy in reproducing blood pressure. Some differences in other hemodynamic metrics were observed between the two methods. Further studies are needed to validate the proposed method by comparing predicted aortic hemodynamic with clinically measured values.

## Data Availability

The original contributions presented in the study are included in the article/Supplementary Material, further inquiries can be directed to the corresponding authors.
