# Supplementary material for: Estimating flow division in aortic branches of diseased aorta: a method for boundary condition specification in CFD analysis
Source: Front Bioeng Biotechnol. 2025 Nov 13;13:1640687. doi: 10.3389/fbioe.2025.1640687 (PMC12657352; doi:10.3389/fbioe.2025.1640687)
Supplement: Supplementary file 1 [file Supplementaryfile1.docx]

Supplementary Material

**Supplementary Material 1: Reconstruction of the aorta**

The model reconstruction based on the acquisition of the CTA data is shown in **Supplementary Figure 1**.


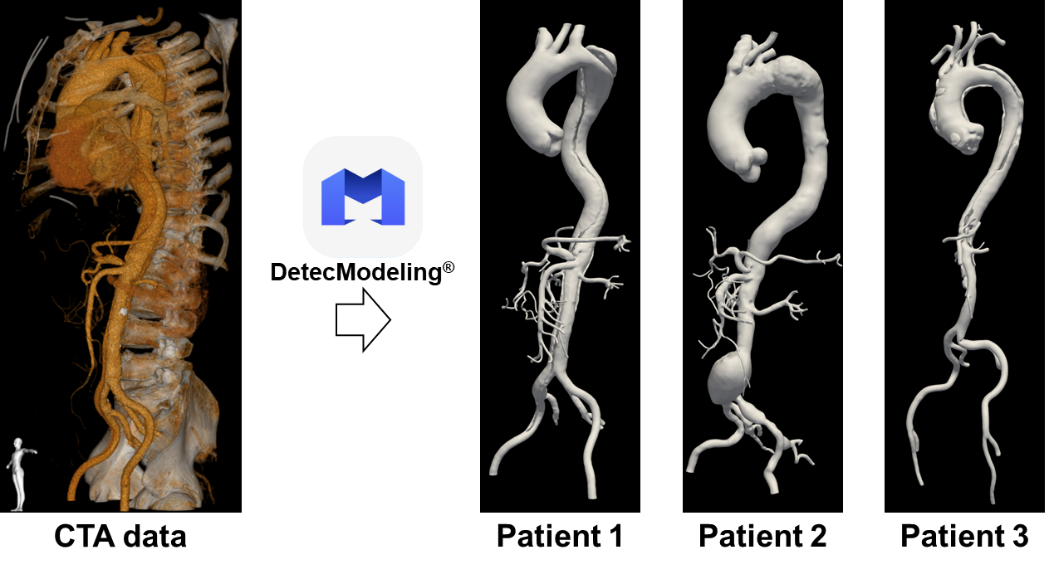


**Supplementary Figure 1. Acquisition of the CTA data and model reconstruction.**

**
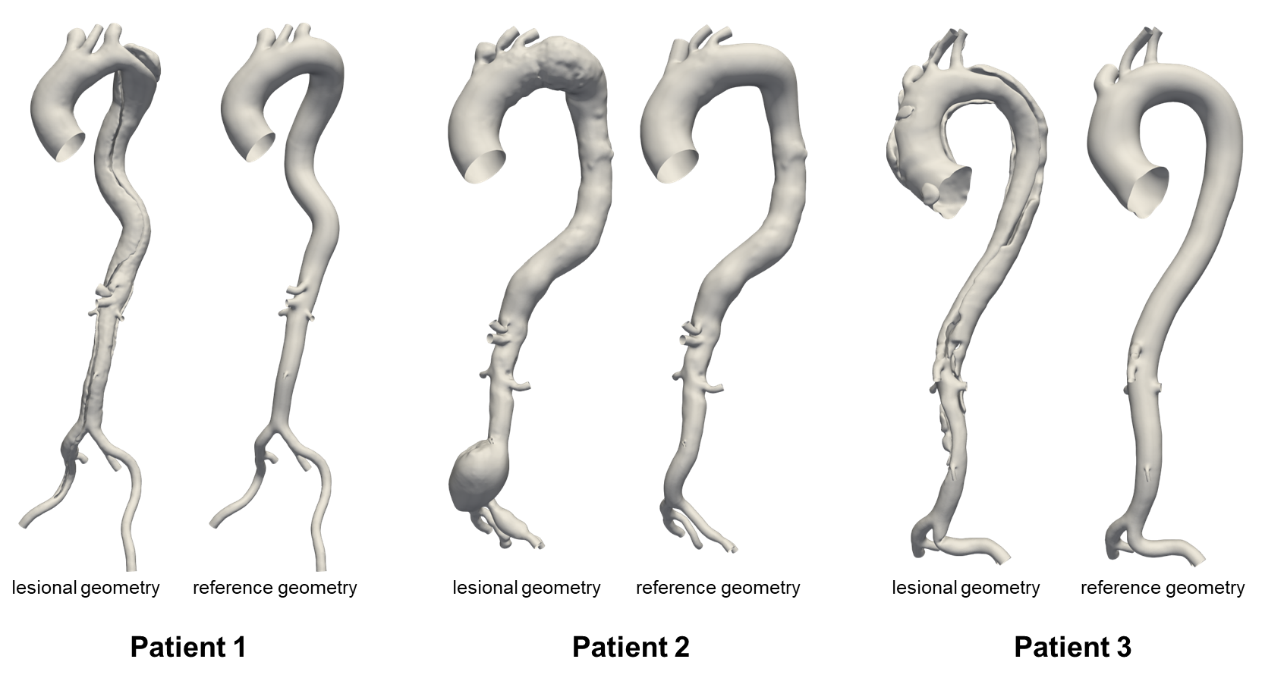
**

**Supplementary Figure 2. Comparison between the lesional geometry and the repaired near-healthy reference geometry.**


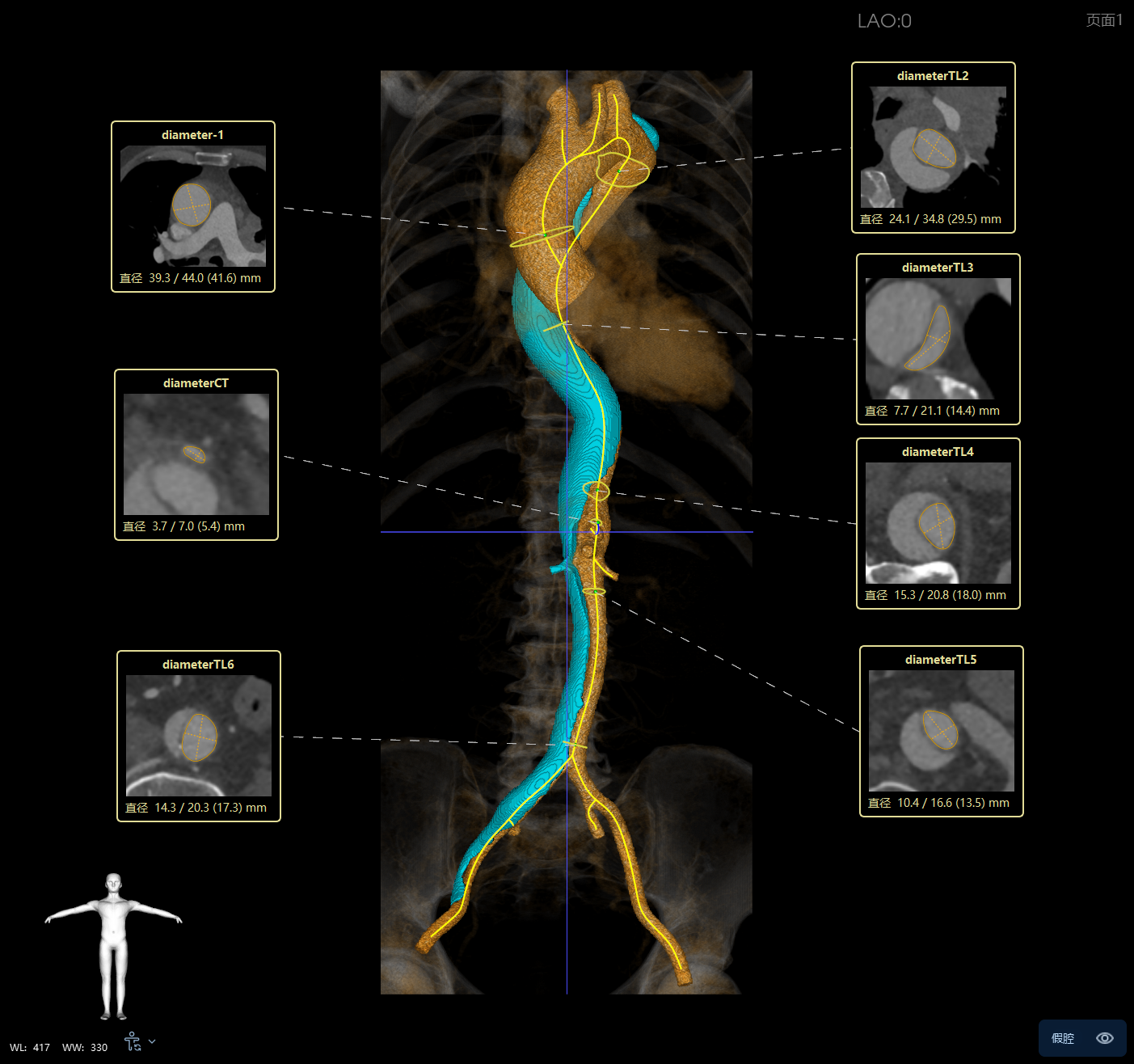


**Supplementary Figure 3. Anatomical measurements of the aortic model in Patient 1, including diameters at clinically relevant regions of interest.**


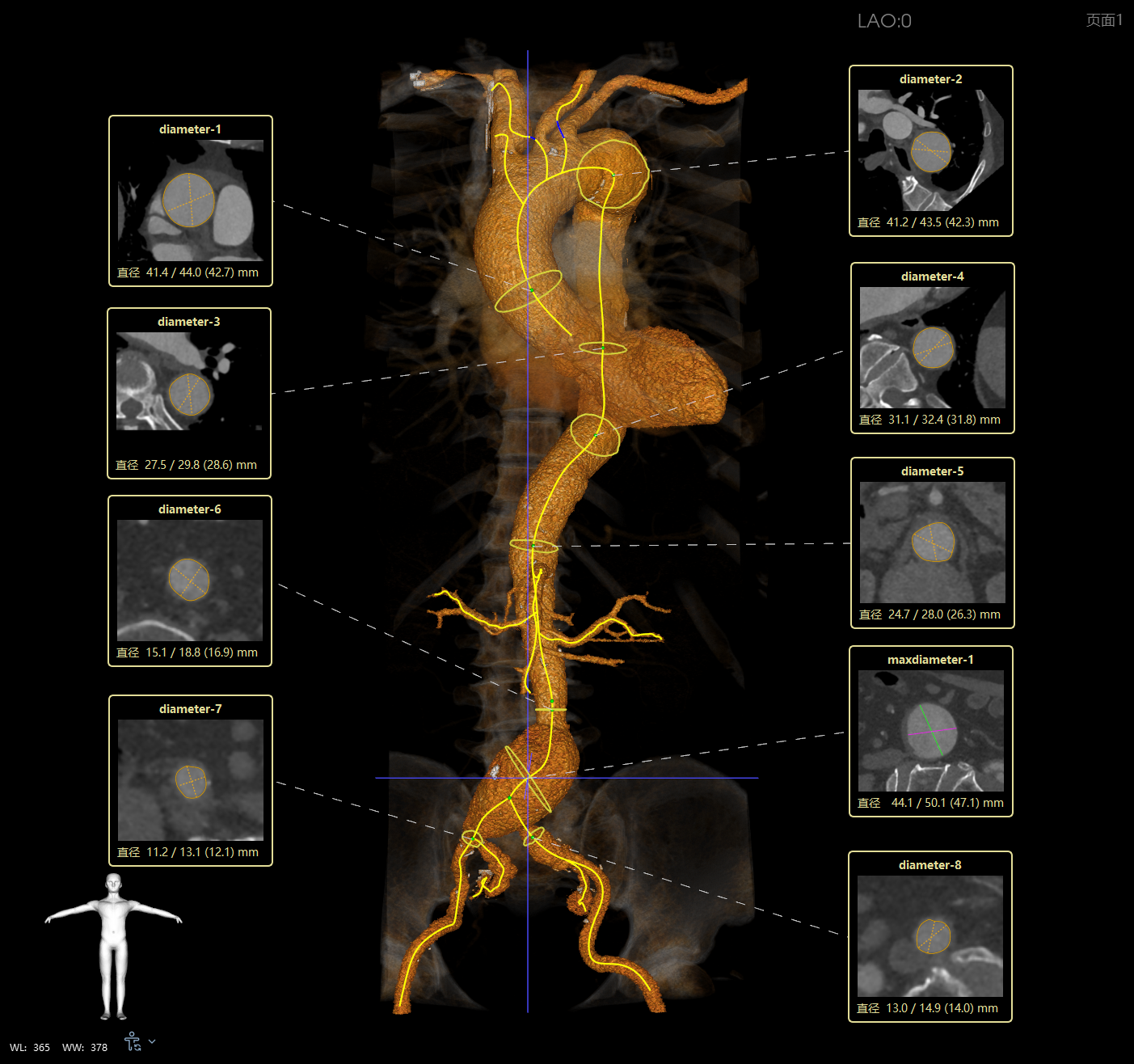


**Supplementary Figure 4. Anatomical measurements of the aortic model in Patient 2, including diameters at clinically relevant regions of interest.**


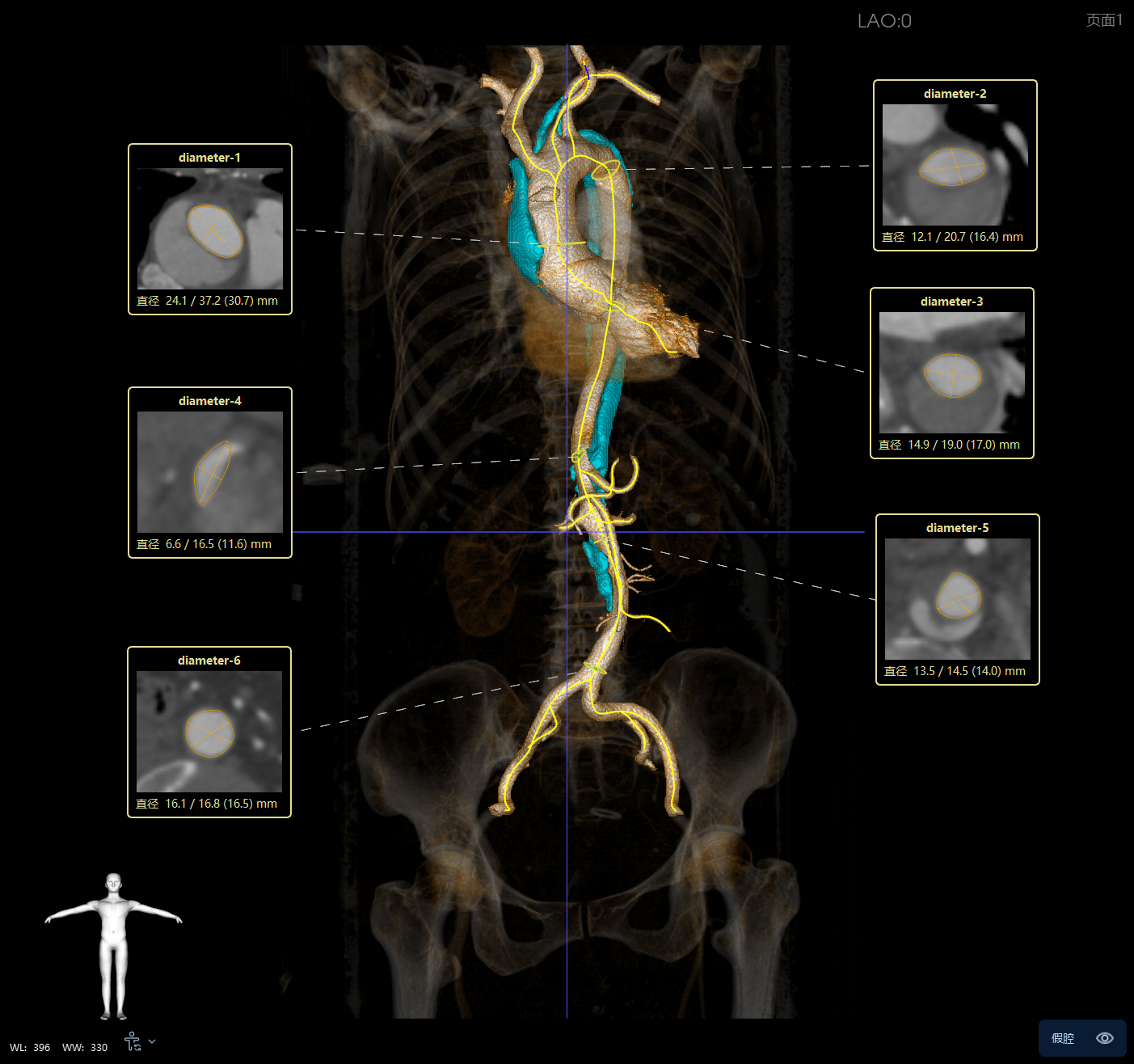


**Supplementary Figure 5. Anatomical measurements of the aortic model in Patient 3, including diameters at clinically relevant regions of interest.**

**Supplementary Material 2: Grid independence check**

To ensure the grid resolution is adequate for accurate CFD predictions, a grid independence check was performed on the aorta of Patient 1. Three grids with varying resolutions were generated for the lesional aorta geometry. Steady-state simulations using the systolic averaged inflow rate were conducted for each grid, with all other settings consistent with those described in **Section 2.7**. The influence of grid resolution on the results was evaluated using the grid convergence index (GCI) (Craven et al., 2009), calculated as follows:

$r_{\text{f,m}}=\left(\frac{N_{\text{f}}}{N_{\text{m}}}\right)^{1/3}$ $r_{\text{m,c}}=\left(\frac{N_{\text{m}}}{N_{\text{c}}}\right)^{1/3}$ (S1)

$r=\frac{r_{\text{f,m}}+r_{\text{m,c}}}{2}$ (S2)

where *r* is the grid refinement factor, *N* is the number of elements in the mesh, the subscripts f, m, and c represent fine, medium, and coarse meshes, respectively.

$p=\frac{\ln\left(\frac{\left|f_{\text{c}}-f_{\text{m}}\right|}{\left|f_{\text{m}}-f_{\text{f}}\right|}\right)}{\ln\left(r\right)}$ (S3)

$E_{\text{f,m}}=\frac{\left(\frac{\left|f_{\text{m}}-f_{\text{f}}\right|}{f_{\text{f}}}\right)}{r^{p}-1}$ $E_{\text{m,c}}=\frac{\left(\frac{\left|f_{\text{c}}-f_{\text{m}}\right|}{f_{\text{m}}}\right)}{r^{p}-1}$ (S4)

$\text{GCI}_{\text{f,m}}=F_{\text{s}}\left|E_{\text{f,m}}\right|$ $\text{GCI}_{\text{m,c}}=F_{\text{s}}\left|E_{\text{m,c}}\right|$ (S5)

where *p* is the observed order of convergence, *F*_s_ is a factor of safety of 1.25, *E* is the estimated percent error.

Mean WSS, mean wall pressure, and mean blood velocity were selected as variables of interest. The mean WSS and mean wall pressure were evaluated over the entire luminal surface of the aortic wall, whereas the mean blood velocity was computed within the flow lumen. The grid properties and grid independence check results are summarized in **Supplementary Table 1**. The GCI values for these variables decreased after grid refinement, indicating reduced dependency of the CFD results on grid resolution. The GCI_f,m_ values were below 2%, demonstrating that the grid resolution was sufficiently fine (Kelsey et al., 2017). Therefore, the medium grid with five boundary layers was chosen for further studies. The grid properties for all models used in this study are shown in **Supplementary Table 2**.

**Supplementary Table 1. Results of grid independence check**

| Metric | Coarse | Medium | Fine | GCI_f,m_ | GCI_m,c_ |
| --- | --- | --- | --- | --- | --- |
| Min. surface size (mm) | 0.8 | 0.3 | 0.1 | —— | |
| Max. surface size (mm) | 1.5 | 1.0 | 0.5 |  |  |
| Global cell size (mm) | 2.5 | 1.5 | 0.8 |  |  |
| Elements | 1599675 | 4579998 | 24152153 |  |  |
| Mean WSS (Pa) | 2.604 | 2.767 | 2.760 | 0.02% | 0.34% |
| Mean wall pressure (Pa) | -213.199 | -198.703 | -194.81 | 0.92% | 3.35% |
| Mean blood velocity (m·s^-1^) | 0.165 | 0.166 | 0.166 | 0.32% | 0.49% |

**Supplementary Table 2. Grid properties for all models in this study**

| Aorta geometry | | Grid properties | | | | |
| --- | --- | --- | --- | --- | --- | --- |
|  |  | Number of elements | | | Minimum orthogonal quality | Maximum skewness |
|  |  | Node | Face | Cell |  |  |
| Patient 1 | Lesional aorta | 917256 | 6055180 | 2658765 | 0.20 | 0.80 |
|  | Repaired aorta | 861293 | 6299420 | 2849903 | 0.21 | 0.80 |
| Patient 2 | Lesional aorta | 973152 | 7279518 | 3312271 | 0.20 | 0.80 |
|  | Repaired aorta | 747620 | 5527472 | 2507461 | 0.20 | 0.80 |
| Patient 3 | Lesional aorta | 1456278 | 9947508 | 4412977 | 0.20 | 0.80 |
|  | Repaired aorta | 752890 | 5526809 | 2502257 | 0.20 | 0.80 |

**Supplementary Material 3: Reproduced outlet flow division and inlet pressure**

**Supplementary Table 3** summarizes the downstream flow division and inlet pressure reproduced by the traditional and proposed methods. The “target” values represent the flow division used to estimate the parameters of the Windkessel model. The “CFD” rows show the outlet flow division and inlet pressure predicted by transient simulations in the traditional and proposed methods. The “RE” rows present the relative error between the predicted and target values.

**Supplementary Table 3. Reproduced downstream flow division and inlet pressure by the traditional and proposed methods.**

| Pat. | Method | Type | Downstream branches flow division (%) | | | | | | | | | | | | Blood pressure (mmHg) | |
| --- | --- | --- | --- | --- | --- | --- | --- | --- | --- | --- | --- | --- | --- | --- | --- | --- |
|  |  |  | BT | LCC | LSA | CT | LRA | RRA | SMA | IMA | LEIA | LIIA | RIIA | REIA | Psys | Pdia |
| 1 | traditional | Target | 17.2 | 4.1 | 8.7 | 15.5 | 10.5 | 10.5 | 10.5 | 0.5 | 7.9 | 3.4 | 3.4 | 7.9 | 134 | 87 |
|  |  | CFD | 16.3 | 3.9 | 8.3 | 20.0 | 9.9 | 9.8 | 9.9 | 0.5 | 7.5 | 3.2 | 3.2 | 7.5 | 140.5 | 47.8 |
|  |  | RE | -5.5% | -5.1% | -4.8% | 22.5% | -6.1% | -7.1% | -6.1% | 0.0% | -5.0% | -5.5% | -5.5% | -5.0% | 4.9% | -45.0% |
|  | proposed | Target | 18.9 | 4.5 | 9.5 | 9.1 | 11.2 | 10.8 | 11.4 | 0.5 | 8.5 | 3.6 | 3.6 | 8.4 | 134 | 87 |
|  |  | CFD | 18.5 | 4.4 | 9.3 | 11.3 | 10.9 | 10.5 | 11.1 | 0.5 | 8.3 | 3.5 | 3.5 | 8.2 | 145.0 | 70.3 |
|  |  | RE | -2.2% | -2.3% | -2.2% | 19.5% | -2.8% | -2.9% | -2.7% | 0.0% | -2.4% | -2.9% | -2.9% | -2.4% | 8.2% | -19.2% |
| 2 | traditional | Target | 22.9 | 3.3 | 3.8 | 15.5 | 10.5 | 10.5 | 10.5 | 0.5 | 7.9 | 3.4 | 3.4 | 7.9 | 113 | 87 |
|  |  | CFD | 22.3 | 3.2 | 3.7 | 17.9 | 10.1 | 10.2 | 10.2 | 0.5 | 7.7 | 3.3 | 3.3 | 7.6 | 118.4 | 82.7 |
|  |  | RE | -2.7% | -3.1% | -2.7% | 13.4% | -4.0% | -2.9% | -2.9% | 0.0% | -2.3% | -2.3% | -2.3% | -3.6% | 4.8% | -5.0% |
|  | proposed | Target | 18.7 | 5.5 | 6.9 | 12.6 | 10.9 | 10.6 | 10.9 | 0.5 | 8.2 | 3.5 | 3.5 | 8.2 | 113 | 87 |
|  |  | CFD | 18.4 | 5.4 | 6.7 | 14.4 | 10.6 | 10.6 | 10.6 | 0.5 | 8.0 | 3.4 | 3.4 | 8.0 | 119.4 | 84.7 |
|  |  | RE | -1.6% | -1.9% | -3.0% | 12.5% | -2.8% | 0.0% | -2.8% | 0.0% | -2.5% | -2.9% | -2.9% | -2.5% | 5.7% | -2.6% |
| 3 | traditional | Target | 18.1 | 6.0 | 5.9 | 15.5 | 10.5 | 10.5 | 10.5 | 0.5 | 7.9 | 3.4 | 3.4 | 7.9 | 129 | 83 |
|  |  | CFD | 18.1 | 6.0 | 5.9 | 15.4 | 10.6 | 11.0 | 10.3 | 0.5 | 7.8 | 3.3 | 3.3 | 7.8 | 153.8 | 72.1 |
|  |  | RE | 0.0% | 0.0% | 0.0% | -0.6% | 0.9% | 4.5% | -1.9% | 0.0% | -1.0% | -2.3% | -2.3% | -1.0% | 34.9% | -22.1% |
|  | proposed | Target | 19.7 | 6.5 | 6.5 | 15.1 | 9.9 | 9.3 | 10.5 | 0.5 | 7.7 | 3.3 | 3.3 | 7.7 | 129 | 83 |
|  |  | CFD | 19.7 | 6.5 | 6.5 | 15.0 | 10.0 | 9.6 | 10.3 | 0.5 | 7.6 | 3.3 | 3.3 | 7.7 | 151.5 | 72.1 |
|  |  | RE | 0.0% | 0.0% | 0.0% | -0.7% | 1.0% | 3.1% | -1.9% | 0.0% | -1.3% | 0.0% | 0.0% | 0.0% | 32.9% | -22.1% |

**Supplementary Material 4: Estimated parameters of the Windkessel model**

First, the parameters of the Windkessel models were estimated using the fast approach proposed by Li and Mao (Li and Mao, 2023) and were designated as “traditional” for differentiation. Next, based on the framework proposed in this study, the flow division of downstream branches was calculated and used to determine the parameters of the Windkessel model. The parameters obtained using this framework were designated as “proposed”. **Supplementary Table 4** summarizes the parameters of the Windkessel models calculated by the traditional and proposed methods.

**Supplementary Table 4. Parameters (*R*_c,_ *R*_p_, and *C*) in the Windkessel model obtained from the traditional and proposed methods.**

| Patient | Branch | *R*_c_ (mmHg·s·ml^-1^) | | *R*_p_ (mmHg·s·ml^-1^) | | *C* (ml·mmHg^-1^) | |
| --- | --- | --- | --- | --- | --- | --- | --- |
|  |  | traditional | proposed | traditional | proposed | traditional | proposed |
| Patient 1  Traditional P_ref_: 5 mmHg  Proposed P_ref_: 56 mmHg | BT | 1.069 | 0.778 | 4.200 | 1.512 | 0.268 | 0.335 |
|  | LCC | 4.504 | 3.237 | 17.596 | 6.377 | 0.063 | 0.078 |
|  | LSA | 2.126 | 1.530 | 8.278 | 3.016 | 0.137 | 0.169 |
|  | CT | 0.350 | 0.557 | 0.679 | 1.048 | 0.085 | 0.047 |
|  | LRA | 1.713 | 1.264 | 6.562 | 2.236 | 0.169 | 0.195 |
|  | RRA | 1.714 | 1.290 | 6.318 | 2.099 | 0.169 | 0.184 |
|  | SMA | 1.760 | 1.262 | 6.735 | 2.403 | 0.168 | 0.200 |
|  | IMA | 36.691 | 29.058 | 141.772 | 54.764 | 0.008 | 0.009 |
|  | LEIA | 2.329 | 1.692 | 8.985 | 3.217 | 0.126 | 0.147 |
|  | LIIA | 5.425 | 4.040 | 21.077 | 7.662 | 0.055 | 0.063 |
|  | RIIA | 5.479 | 3.992 | 20.964 | 7.645 | 0.054 | 0.061 |
|  | REIA | 2.343 | 1.712 | 8.932 | 3.213 | 0.124 | 0.148 |
| Patient 2  Traditional P_ref_: 70 mmHg  Proposed P_ref_: 75 mmHg | BT | 0.285 | 0.345 | 1.103 | 1.025 | 1.065 | 0.835 |
|  | LCC | 1.974 | 1.173 | 7.663 | 3.460 | 0.154 | 0.244 |
|  | LSA | 1.716 | 0.934 | 6.659 | 2.766 | 0.177 | 0.307 |
|  | CT | 0.239 | 0.199 | 0.570 | 0.787 | 0.287 | 0.182 |
|  | LRA | 0.620 | 0.588 | 2.242 | 1.589 | 0.484 | 0.472 |
|  | RRA | 0.617 | 0.596 | 1.990 | 1.394 | 0.474 | 0.439 |
|  | SMA | 0.622 | 0.590 | 2.350 | 1.700 | 0.487 | 0.481 |
|  | IMA | 13.037 | 12.874 | 49.301 | 37.041 | 0.023 | 0.022 |
|  | LEIA | 0.828 | 0.785 | 3.117 | 2.241 | 0.365 | 0.361 |
|  | LIIA | 1.934 | 1.837 | 7.255 | 5.238 | 0.156 | 0.154 |
|  | RIIA | 1.933 | 1.842 | 7.393 | 5.374 | 0.157 | 0.155 |
|  | REIA | 0.828 | 0.784 | 3.115 | 2.239 | 0.364 | 0.361 |
| Patient 3  Traditional P_ref_: 70 mmHg  Proposed P_ref_: 70 mmHg | BT | 0.332 | 0.385 | 1.022 | 0.857 | 0.096 | 0.123 |
|  | LCC | 0.978 | 1.110 | 3.057 | 2.601 | 0.032 | 0.039 |
|  | LSA | 1.200 | 1.301 | 2.935 | 2.443 | 0.035 | 0.045 |
|  | CT | 0.365 | 0.352 | 0.690 | 0.760 | 0.091 | 0.084 |
|  | LRA | 0.476 | 0.554 | 0.980 | 1.057 | 0.056 | 0.058 |
|  | RRA | 1.177 | 1.235 | 0.000 | 0.246 | 0.577 | 0.022 |
|  | SMA | 0.488 | 0.411 | 1.239 | 1.346 | 0.057 | 0.052 |
|  | IMA | 10.004 | 12.411 | 27.228 | 25.235 | 0.003 | 0.003 |
|  | LEIA | 0.495 | 0.541 | 1.861 | 1.898 | 0.038 | 0.038 |
|  | LIIA | 1.283 | 1.183 | 4.204 | 4.494 | 0.017 | 0.016 |
|  | RIIA | 1.231 | 1.216 | 4.271 | 4.475 | 0.017 | 0.016 |
|  | REIA | 0.545 | 0.505 | 1.827 | 1.948 | 0.039 | 0.037 |

**Supplementary Material 5:** **Hemodynamic results**

The distributions of blood pressure on the selected planes in Patients 2 and 3 at five time points are shown in **Supplementary Figure 3** and **4**, respectively.


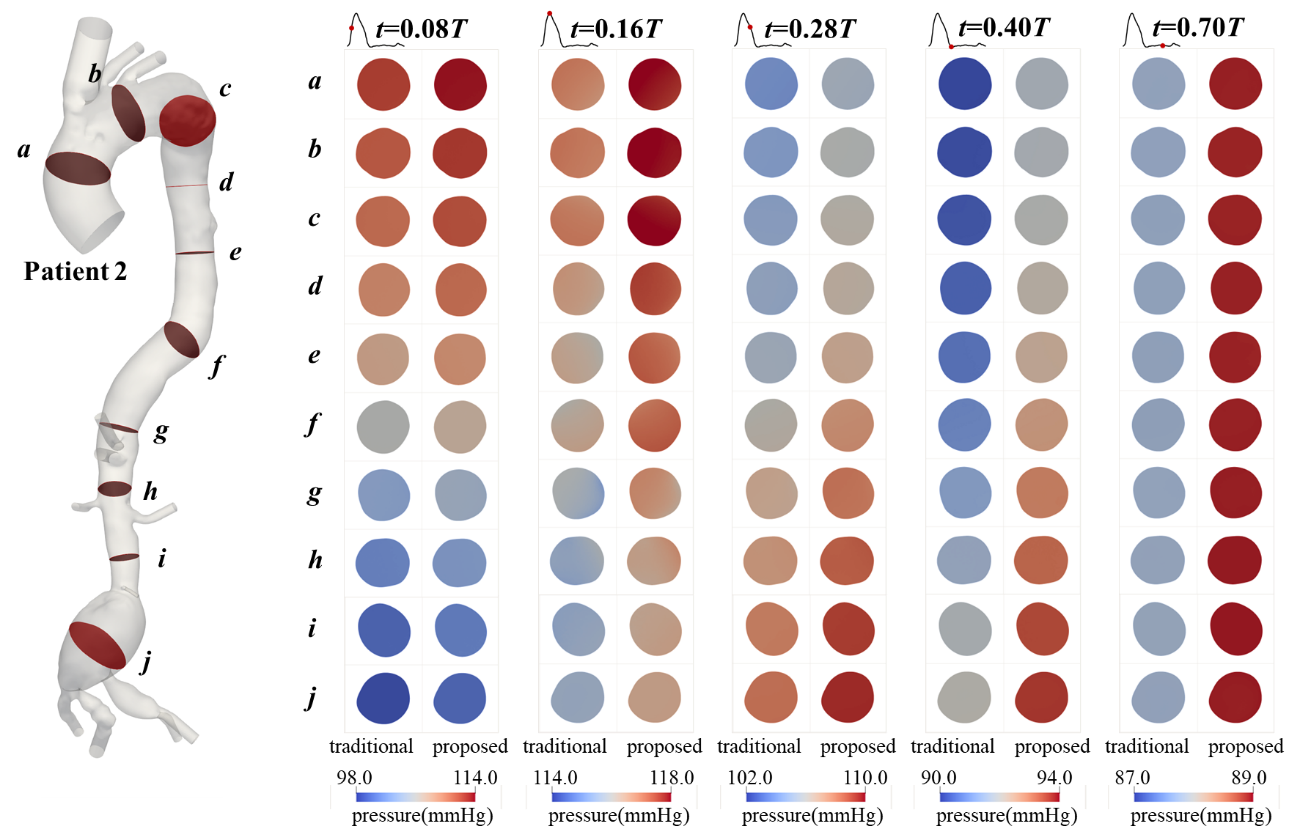


**Supplementary Figure 3. Contours of blood pressure on the selected planes in Patient 2 at mid-systolic acceleration, peak systole, mid-systolic deceleration, early diastole, and mid-diastole.**


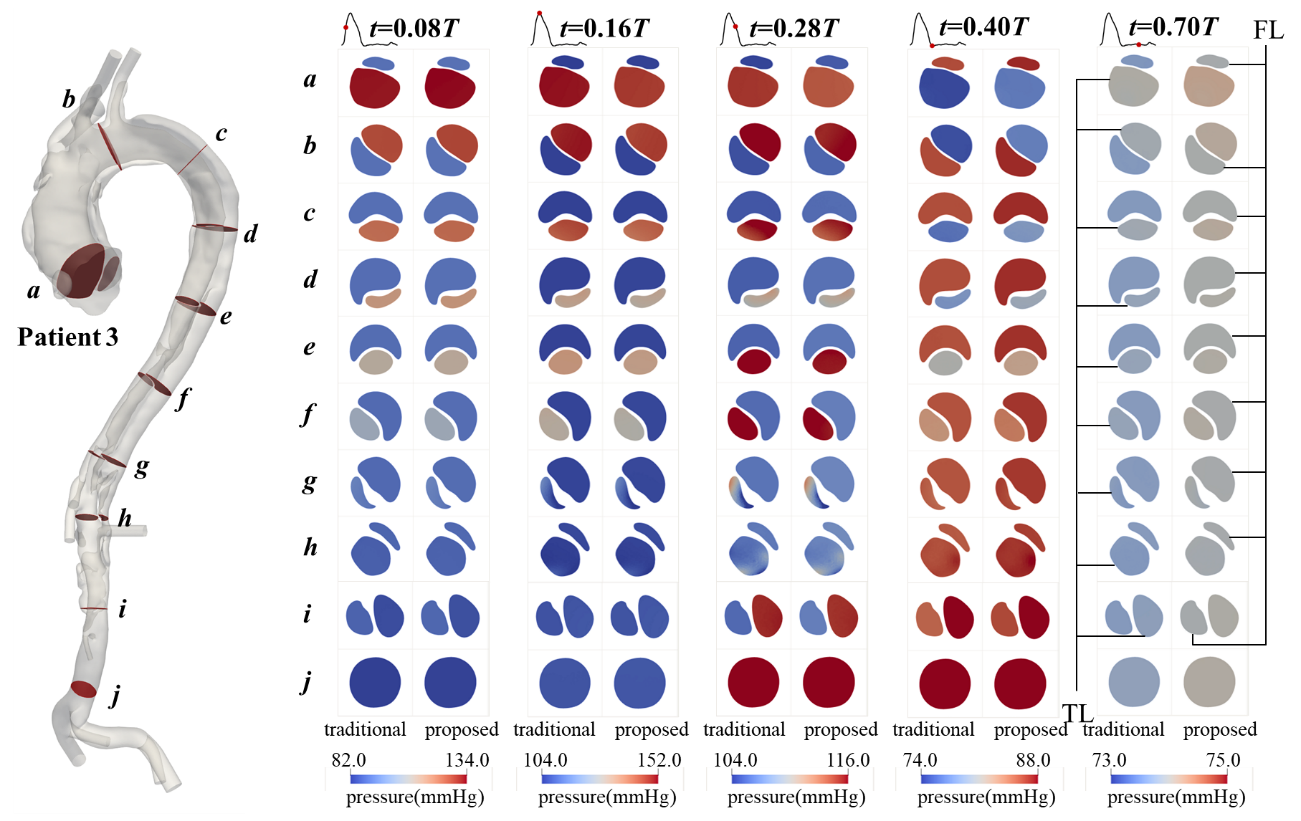


**Supplementary Figure 4. Contours of blood pressure on the selected planes in Patient 3 at mid-systolic acceleration, peak systole, mid-systolic deceleration, early diastole, and mid-diastole.**

The velocity streamlines in Patient 2 and 3 at five time points are shown in **Supplementary Figure 5** and **S6**, respectively.


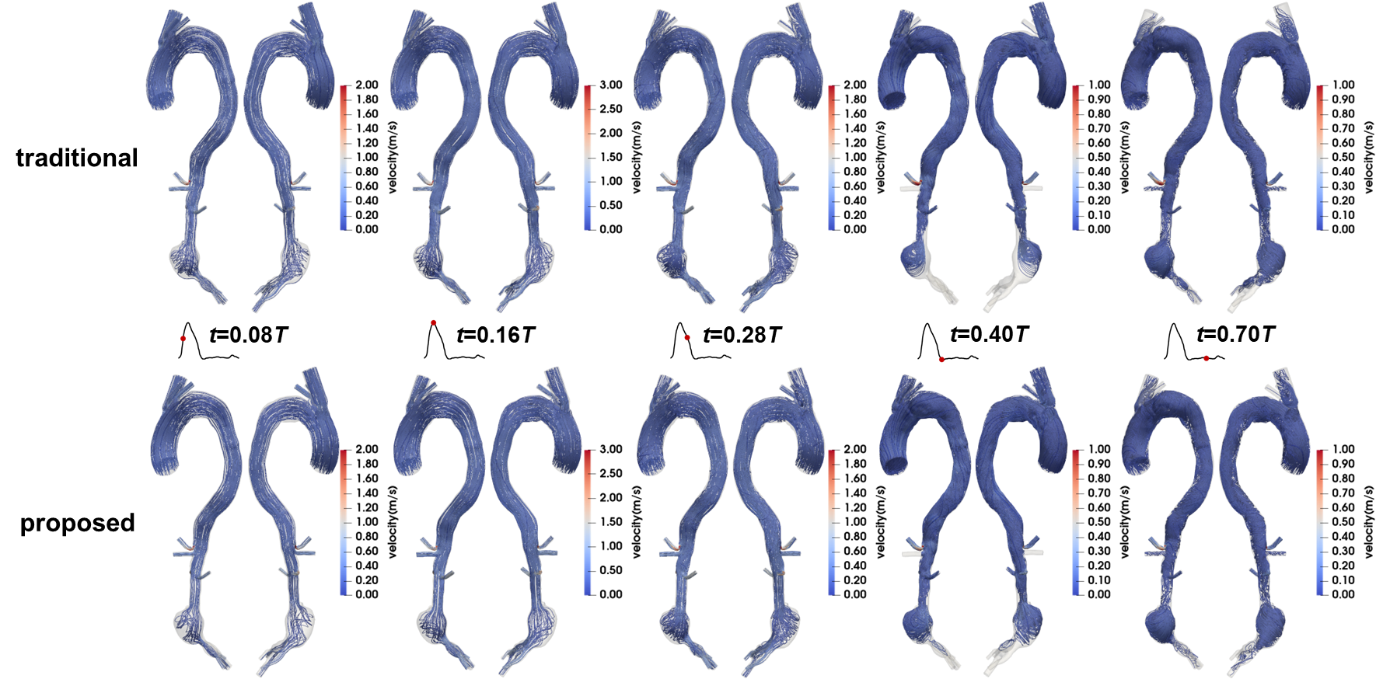


**Supplementary Figure 5. Velocity streamlines in Patient 2 at mid-systolic acceleration, peak systole, mid-systolic deceleration, early diastole, and mid-diastole.**


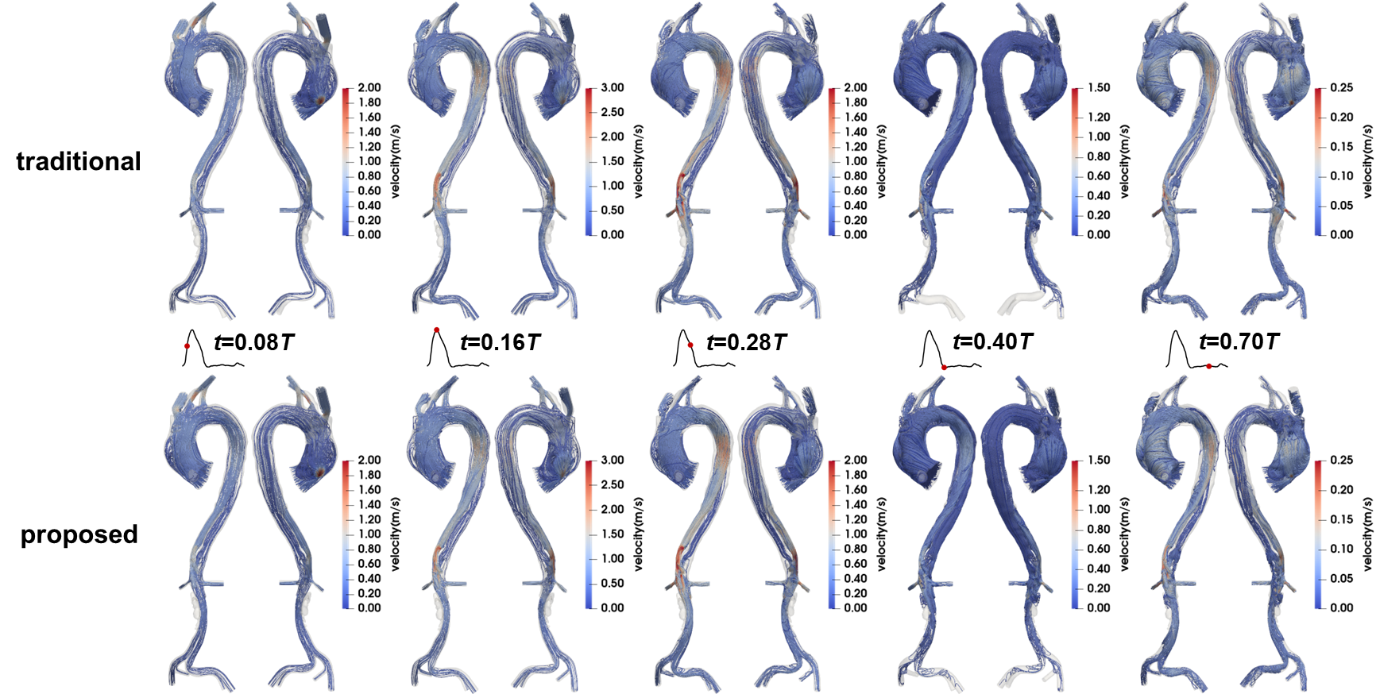


**Supplementary Figure 6. Velocity streamlines in Patient 3 at mid-systolic acceleration, peak systole, mid-systolic deceleration, early diastole, and mid-diastole.**

The distributions of velocity on the selected planes in Patients 2 and 3 at five time points are shown in **Supplementary Figure 7** and **8**, respectively.


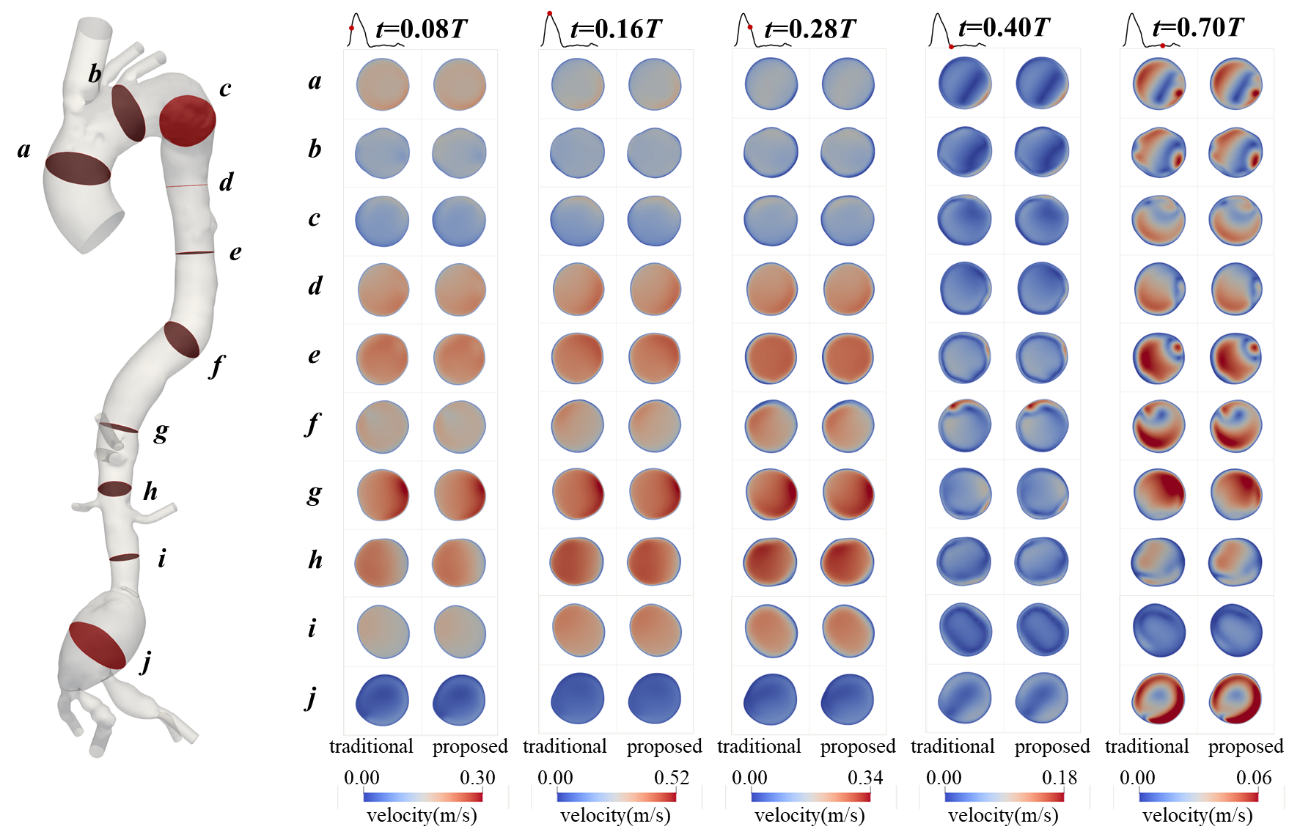


**Supplementary Figure 7. Contours of blood velocity on the selected planes in Patient 2 at mid-systolic acceleration, peak systole, mid-systolic deceleration, early diastole, and mid-diastole.**


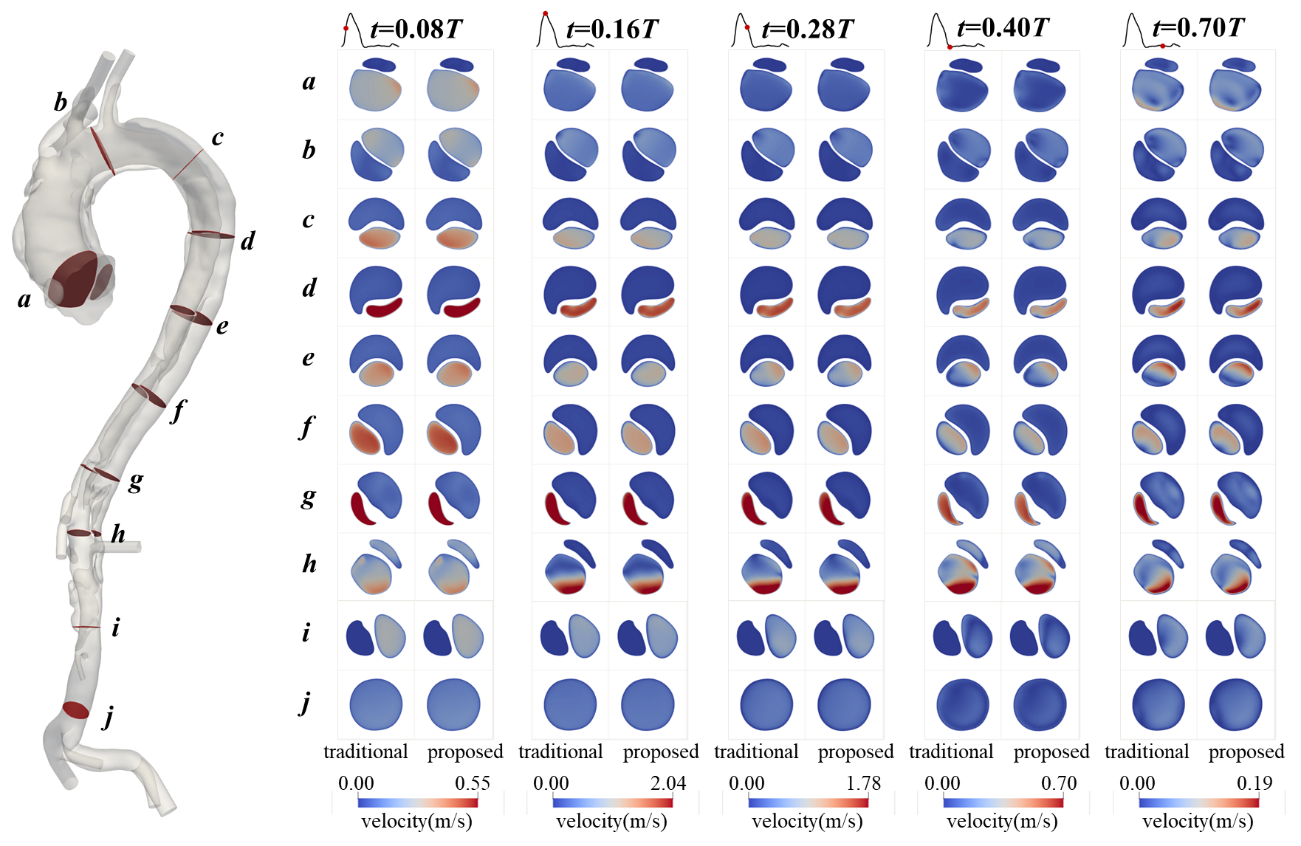


**Supplementary Figure 8. Contours of blood velocity on the selected planes in Patient 3 at mid-systolic acceleration, peak systole, mid-systolic deceleration, early diastole, and mid-diastole.**

The distributions of local normalized helicity on the selected planes in Patients 1, 2 and 3 at five time points are shown in **Supplementary Figure 9**, **10** and **11**, respectively.


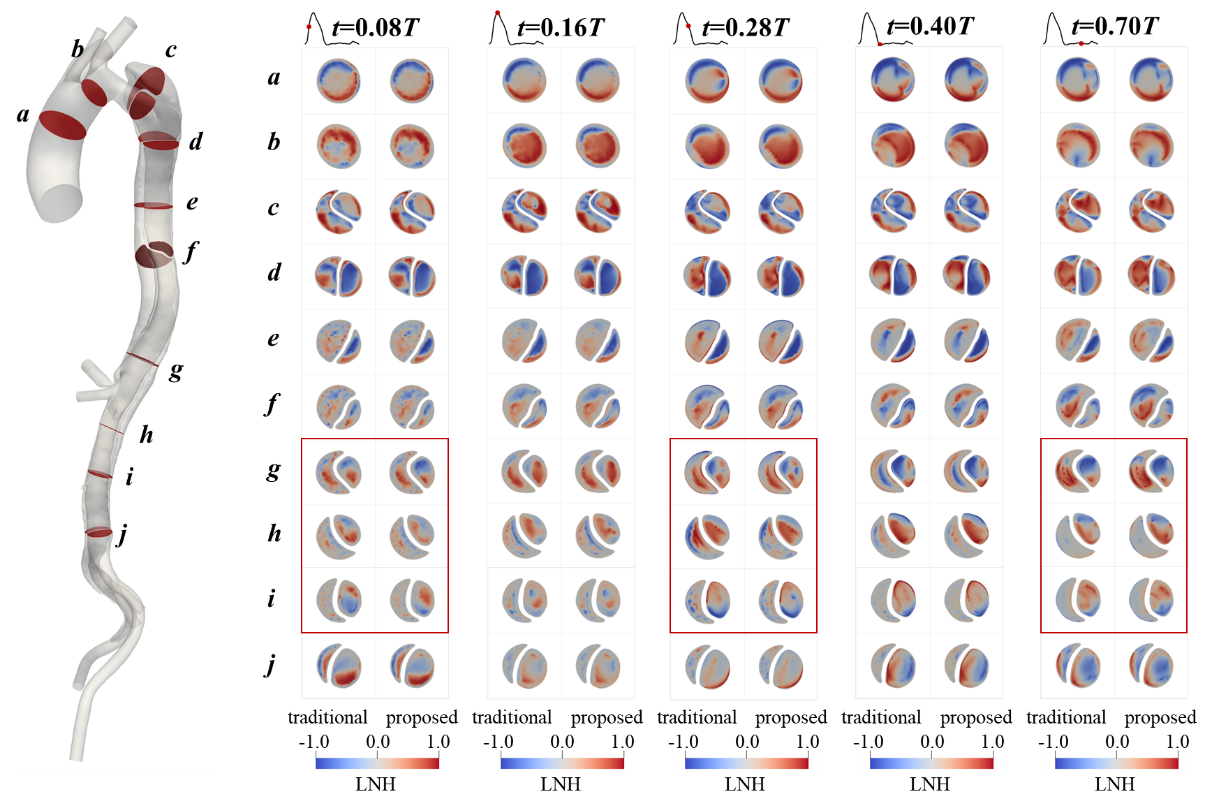


**Supplementary Figure 9. Contours of local normalized helicity on the selected planes in Patient 1 at mid-systolic acceleration, peak systole, mid-systolic deceleration, early diastole, and mid-diastole.**


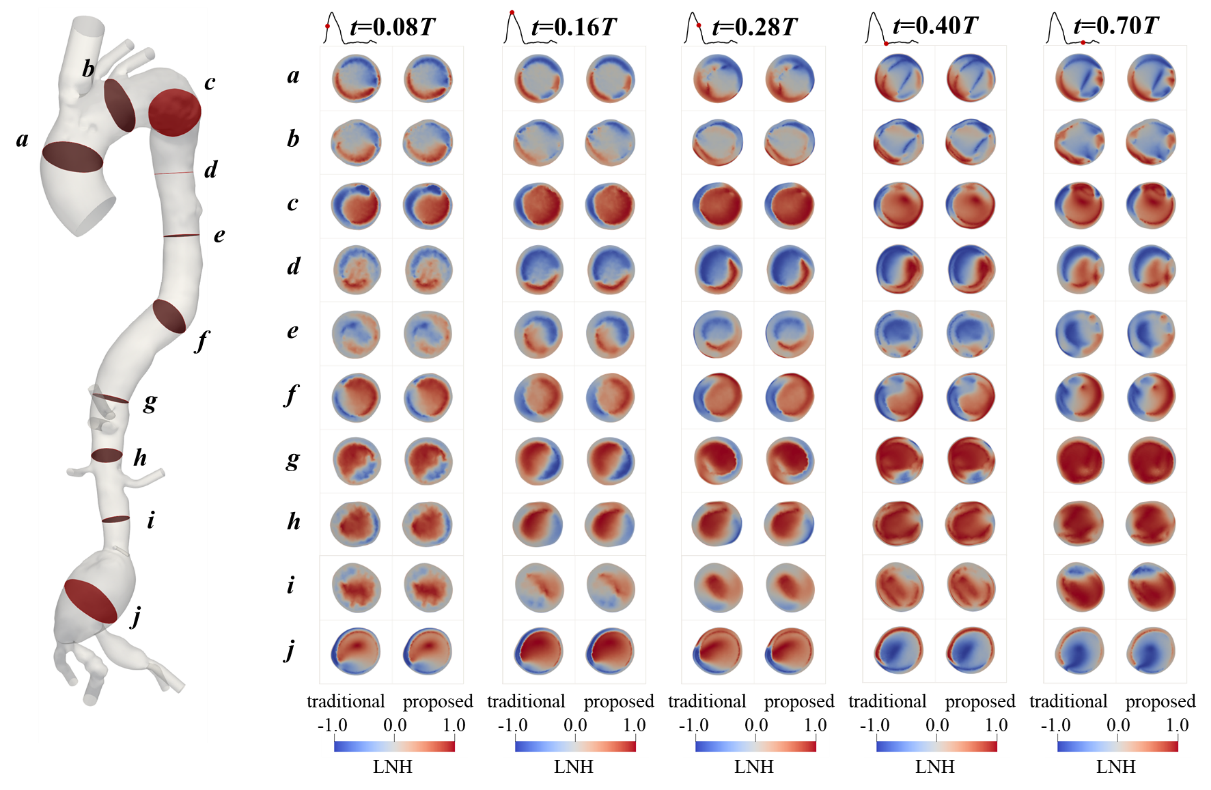


**Supplementary Figure 10. Contours of local normalized helicity on the selected planes in Patient 2 at mid-systolic acceleration, peak systole, mid-systolic deceleration, early diastole, and mid-diastole.**


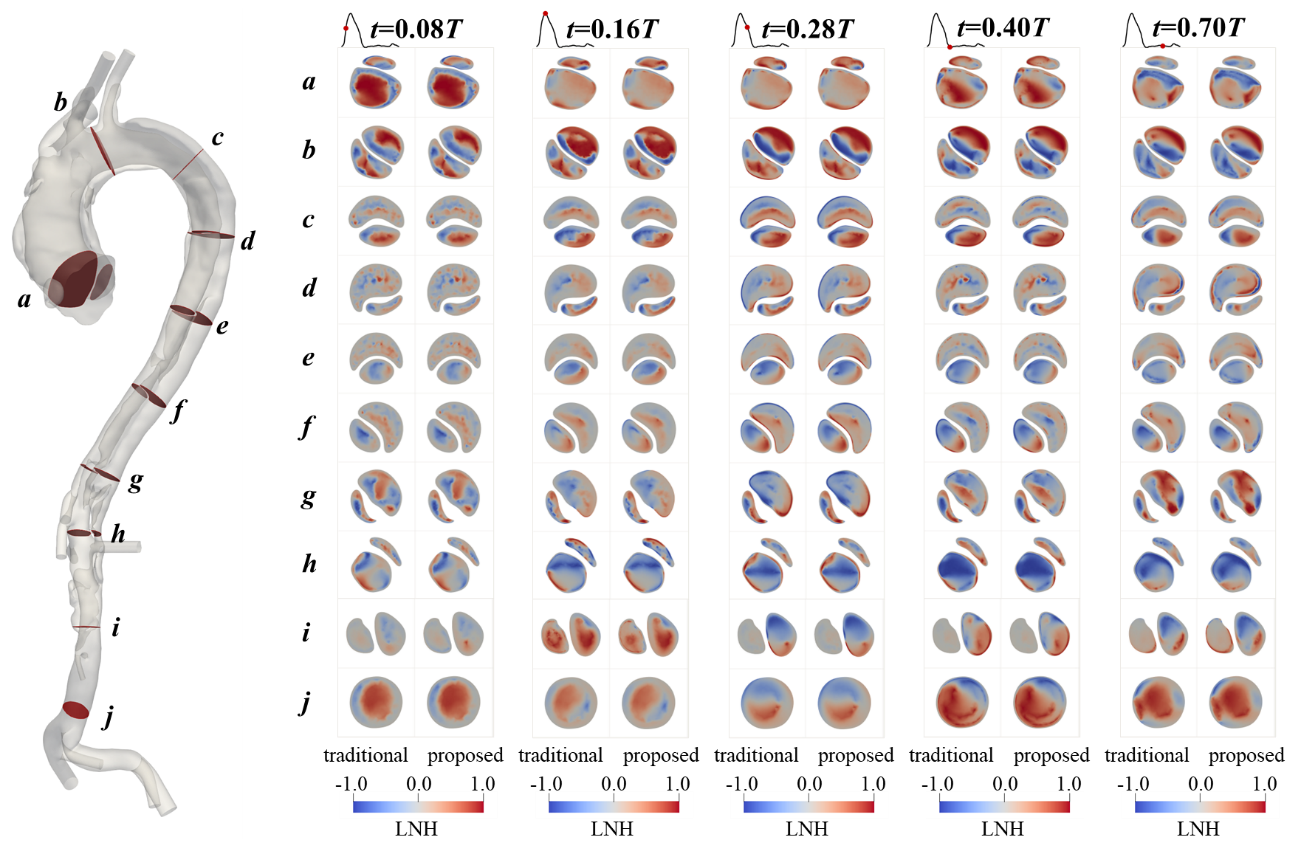


**Supplementary Figure 11. Contours of local normalized helicity on the selected planes in Patient 3 at mid-systolic acceleration, peak systole, mid-systolic deceleration, early diastole, and mid-diastole.**

The difference in local normalized helicity predicted by the proposed and traditional methods in Patients 2 and 3 are shown in **Supplementary Figure 12** and **13**.


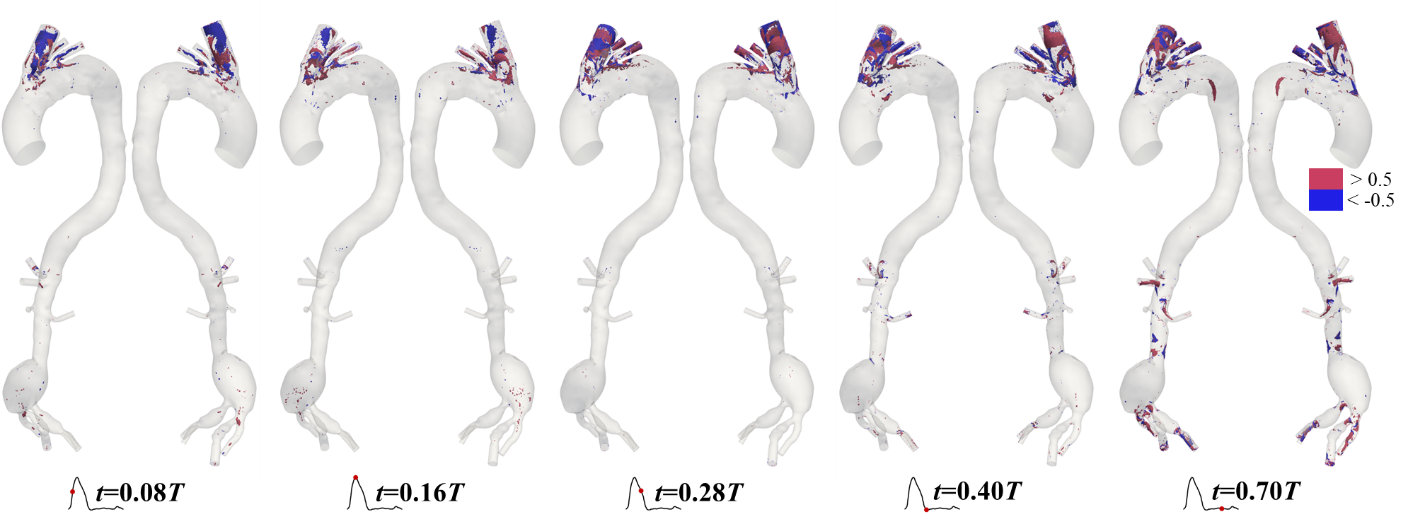


**Supplementary Figure 12. Differences in local normalized helicity in Patient 2 at mid-systolic acceleration, peak systole, mid-systolic deceleration, early diastole, and mid-diastole.**


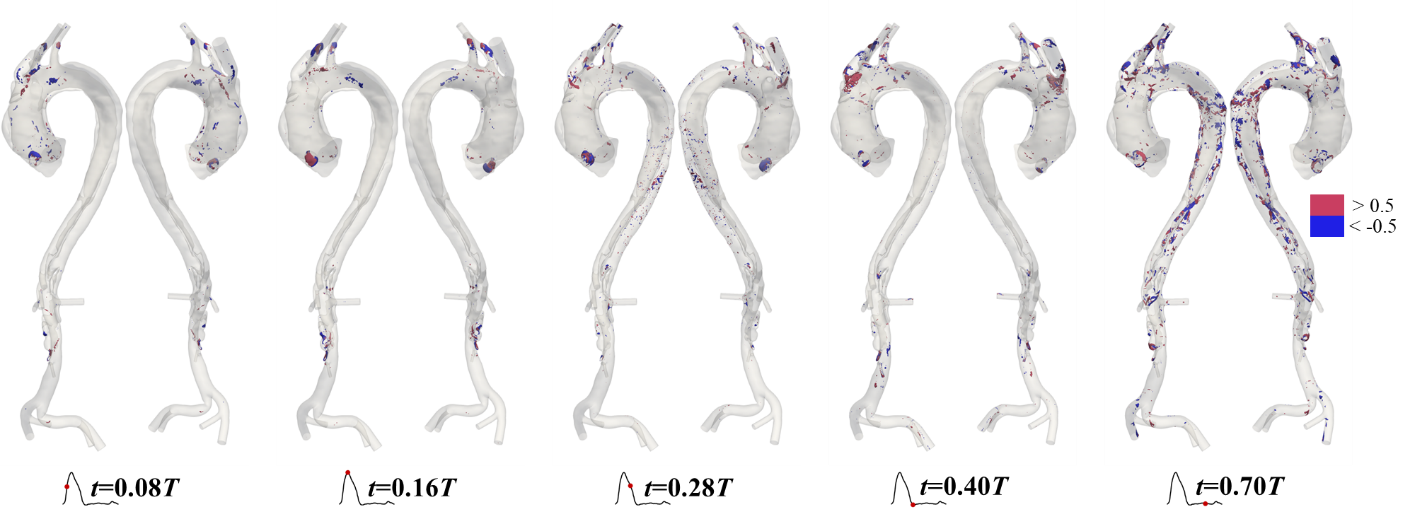


**Supplementary Figure 13. Differences in local normalized helicity in Patient 3 at mid-systolic acceleration, peak systole, mid-systolic deceleration, early diastole, and mid-diastole.**

The distributions and differences of WSS-related metrics in Patients 2 and 3 predicted by the traditional and proposed methods are shown in **Supplementary Figure 14** and **15**.


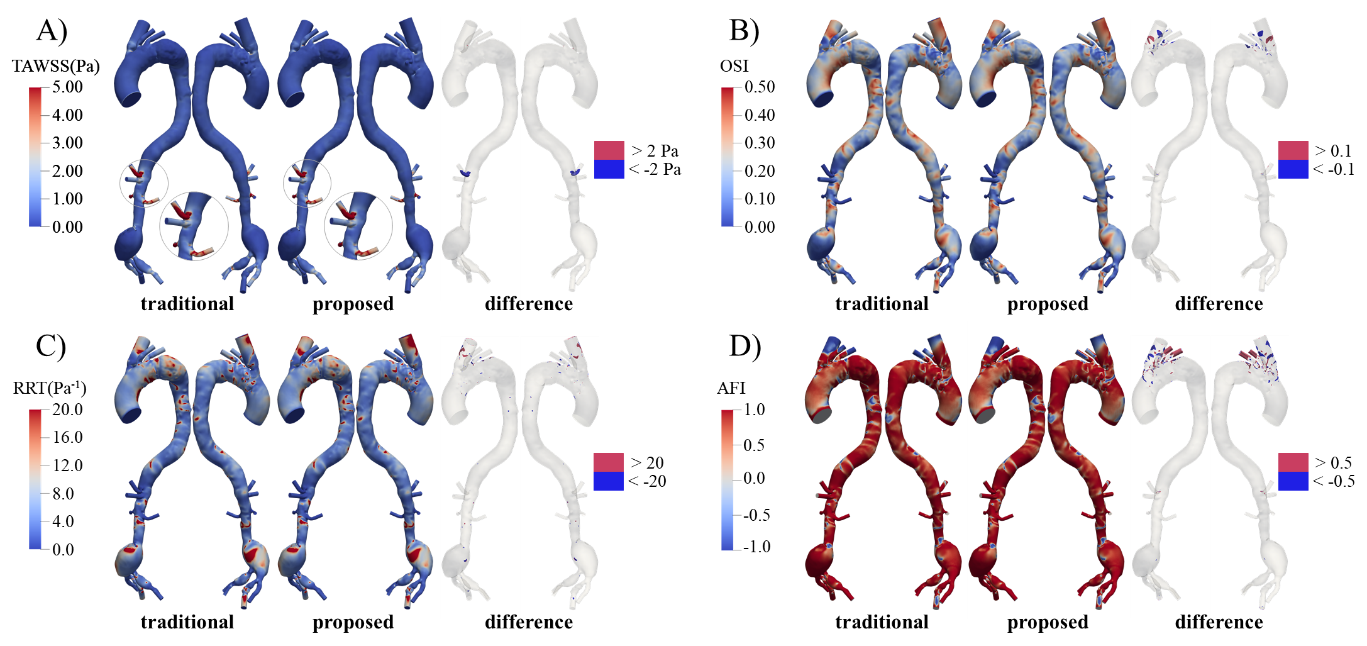


**Supplementary Figure 14. Distribution and difference of WSS-related metrics in Patient 2 predicted by the traditional and proposed methods. A) TAWSS; B) OSI; C) RRT; D) AFI.**


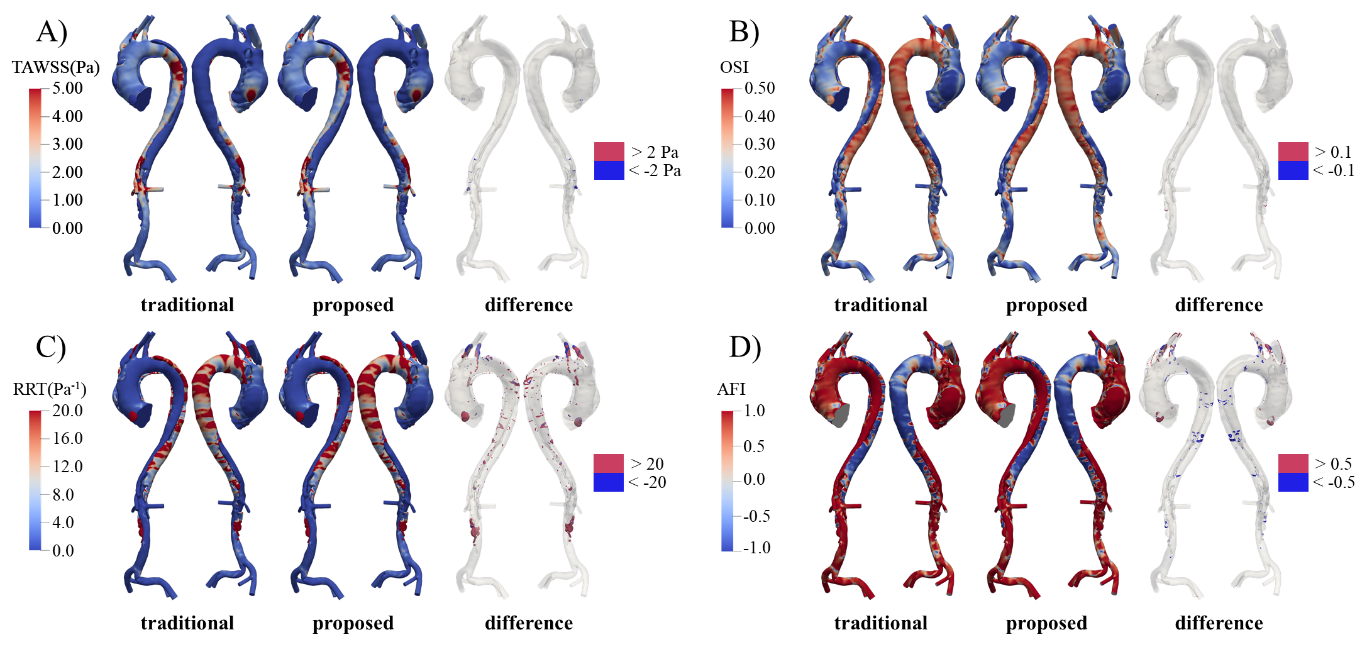


**Supplementary Figure 15. Distribution and difference of WSS-related metrics in Patient 3 predicted by the traditional and proposed methods. A) TAWSS; B) OSI; C) RRT; D) AFI.**

**References**

Craven, B. A., Paterson, E. G., Settles, G. S., and Lawson, M. J. (2009). Development and Verification of a High-Fidelity Computational Fluid Dynamics Model of Canine Nasal Airflow. *Journal of Biomechanical Engineering* 131, 091002. doi: 10.1115/1.3148202

Kelsey, L. J., Powell, J. T., Norman, P. E., Miller, K., and Doyle, B. J. (2017). A comparison of hemodynamic metrics and intraluminal thrombus burden in a common iliac artery aneurysm. *Int J Numer Method Biomed Eng* 33. doi: 10.1002/cnm.2821

Li, Z., and Mao, W. (2023). A fast approach to estimating Windkessel model parameters for patient-specific multi-scale CFD simulations of aortic flow. *Computers & Fluids* 259, 105894. doi: 10.1016/j.compfluid.2023.105894
